# Metabolic signatures across the full spectrum of non-alcoholic fatty liver disease

**DOI:** 10.1016/j.jhepr.2022.100477

**Published:** 2022-03-26

**Authors:** Aidan J. McGlinchey, Olivier Govaere, Dawei Geng, Vlad Ratziu, Michael Allison, Jerome Bousier, Salvatore Petta, Claudia de Oliviera, Elisabetta Bugianesi, Jörn M. Schattenberg, Ann K. Daly, Tuulia Hyötyläinen, Quentin M. Anstee, Matej Orešič

**Affiliations:** 1 School of Medical Sciences, Örebro University, Örebro, Sweden; 2 Translational and Clinical Research Institute, Faculty of Medical Sciences, Newcastle University, Newcastle upon Tyne, UK; 3 Department of Chemistry, Örebro University, Örebro, Sweden; 4 Assistance Publique-Hôpitaux de Paris, Hôpital Beaujon, University Paris-Diderot, Paris, France; 5 Liver Unit, Department of Medicine, Cambridge Biomedical Research Centre, Cambridge University NHS Foundation Trust, Cambridge, UK; 6 Hepato-Gastroenterology Department, Angers University Hospital, Angers, France; 7 Dipartimento Biomedico di Medicina Interna e Specialistica Di.Bi.M.I.S, University of Palermo, Palermo, Italy; 8 Universidade de Sao Paulo, Sao Paulo, Sao Paulo, Brazil; 9 Department of Medical Sciences, Division of Gastro-Hepatology, A.O. Città della Salute e della Scienza di Torino, University of Turin, Turin, Italy; 10 Department of Medicine, University Hospital Mainz, Mainz, Germany; 11 Newcastle NIHR Biomedical Research Centre, Newcastle upon Tyne Hospitals, NHS Foundation Trust, Newcastle upon Tyne, UK; 12 Turku Bioscience, University of Turku and Åbo Akademi University, Turku, Finland

**Keywords:** Fibrosis, Lipidomics, Mass spectrometry, Metabolomics, Non-alcoholic steatohepatitis, 2-HB, 2-hydroxybutanoic acid, 3-HB, 3-hydroxybutanoic acid, ALT, alanine aminotransferase, AST, aspartate aminotransferase, CE, cholesterol ester, Cer, ceramide, FFA, free fatty acid, FLIP, Fatty Liver Inhibition of Progression, GC, gas chromatography, HCC, hepatocellular carcinoma, HSD, honest significant difference, LC, lipid cluster, LDL, low-density lipoprotein, LM, lipid and metabolite, LMC, lipid, metabolite, and clinical variable, LPC, lysophosphatidylcholine, NAFL, non-alcoholic fatty liver, NAFLD, non-alcoholic fatty liver disease, NAS, NASH activity score, NASH, non-alcoholic steatohepatitis, NIDDK NASH-CRN, National Institute of Digestive Diseases and Kidney NASH Clinical Research Network, NRR, non-rejection rate, PC, phosphatidylcholine, PCA, principal component analysis, PC(O), ether PC, PE, phosphatidylethanolamine, QTOFMS, quadrupole-time-of-flight mass spectrometry, ROC, receiving operator characteristic, SAF, steatosis, activity, and fibrosis, SM, sphingomyelin, T2DM, type 2 diabetes mellitus, TG, triacylglycerol, UHPLC, ultrahigh-performance liquid chromatography

## Abstract

**Background & Aims:**

Non-alcoholic fatty liver disease (NAFLD) is a progressive liver disease with potentially severe complications including cirrhosis and hepatocellular carcinoma. Previously, we have identified circulating lipid signatures associating with liver fat content and non-alcoholic steatohepatitis (NASH). Here, we develop a metabolomic map across the NAFLD spectrum, defining interconnected metabolic signatures of steatosis (non-alcoholic fatty liver, NASH, and fibrosis).

**Methods:**

We performed mass spectrometry analysis of molecular lipids and polar metabolites in serum samples from the European NAFLD Registry patients (n = 627), representing the full spectrum of NAFLD. Using various univariate, multivariate, and machine learning statistical approaches, we interrogated metabolites across 3 clinical perspectives: steatosis, NASH, and fibrosis.

**Results:**

Following generation of the NAFLD metabolic network, we identify 15 metabolites unique to steatosis, 18 to NASH, and 15 to fibrosis, with 27 common to all. We identified that progression from F2 to F3 fibrosis coincides with a key pathophysiological transition point in disease natural history, with n = 73 metabolites altered.

**Conclusions:**

Analysis of circulating metabolites provides important insights into the metabolic changes during NAFLD progression, revealing metabolic signatures across the NAFLD spectrum and features that are specific to NAFL, NASH, and fibrosis. The F2–F3 transition marks a critical metabolic transition point in NAFLD pathogenesis, with the data pointing to the pathophysiological importance of metabolic stress and specifically oxidative stress.

**Clinical Trials registration:**

The study is registered at Clinicaltrials.gov (NCT04442334).

**Lay summary:**

Non-alcoholic fatty liver disease is characterised by the build-up of fat in the liver, which progresses to liver dysfunction, scarring, and irreversible liver failure, and is markedly increasing in its prevalence worldwide. Here, we measured lipids and other small molecules (metabolites) in the blood with the aim of providing a comprehensive molecular overview of fat build-up, liver fibrosis, and diagnosed severity. We identify a key metabolic ‘watershed’ in the progression of liver damage, separating severe disease from mild, and show that specific lipid and metabolite profiles can help distinguish and/or define these cases.

## Highlights

• We assembled a highly phenotyped and characterised cohort of patients with NAFLD across the full spectrum of NAFLD.
• Lipidomic and metabolomic interrogation of this dataset reveals crucial metabolic tipping point of NAFLD at fibrosis stage F2–F3.
• NAFLD processes of fibrosis, steatosis, and NASH progression parallel distinct changes in metabolomic profiles.
• Oxidative stress-buffering potential of the liver via ether lipids appears 1 of the key changes *en route* to late-stage NASH.

## Graphical abstract

**Figure:**
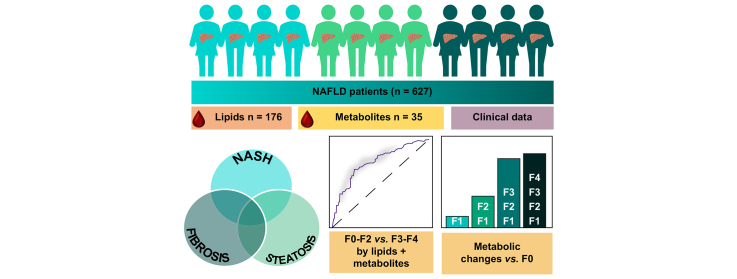

## Introduction

Non-alcoholic fatty liver disease (NAFLD) is a chronic disease, characterised by hepatic triglyceride accumulation exceeding ∼5%, progressing in some to non-alcoholic steatohepatitis (NASH) and advanced fibrosis/cirrhosis.^1^ The world’s leading cause of chronic liver disease, NAFLD has been estimated to affect ∼25% of the adult population and continues to rise with increasing rates of obesity.^2^

NAFLD is associated with several comorbidities including components of metabolic syndrome (type 2 diabetes mellitus [T2DM], hypertension, and dyslipidaemia), cardiovascular disease, end-stage liver disease, and hepatocellular carcinoma (HCC). Given the intricate links between NAFLD and wider metabolic diseases, NAFLD should be considered part of a broad multisystem disease state.^3^ Indeed, the majority of deaths in patients with NAFLD arise owing to cardiovascular disease, which NAFLD exacerbates.^4^

Histological assessment remains the reference standard for assessing NAFLD.^5^ However, liver biopsy is invasive, with small but appreciable risk. Development of non-invasive biomarkers to assess NAFLD severity, whether distinguishing steatosis from NASH or staging fibrosis, remains an unmet clinical need. Lipidomics, the study of lipids in health and disease, forms a sub-part of metabolomics, the study of small (<∼1,500 kDa) molecules in living systems, given their myriad functions as, among others, reactants, substrates, intermediates, and signalling molecules. In this study, we analysed both lipids and polar metabolites, as polar metabolites (*e.g.* amino acids and intermediates in energy metabolism) are those involved in primary, central metabolism and thus of high relevance to the liver metabolism and NAFLD.

The scientific understanding of NAFLD has improved in recent years regarding pathophysiology, genetics, transcriptomics, and metabolomics (including lipidomics).[6], [7], [8] From the lipidomics point of view, recent progress has identified several key changes in the circulating lipidome that accompany progression of NAFLD with evidence converging on specific signatures.[8], [9], [10], [11], [12], [13], [14] Signatures include elevated triacylglycerols (TGs) of low carbon number and double-bond count, specific saturated and monounsaturated free fatty acids (FFAs), ceramides (Cers), and specific phosphatidylcholines (PCs). Nevertheless, studies covering large populations of patients with NAFLD, representing the broad spectrum of NAFLD, that is, from steatosis, through different stages of fibrosis and grades of NASH, are scarce. Here, with the aim of deriving molecular signatures across the full spectrum of NAFLD, we performed comprehensive lipidomics and targeted analyses of polar metabolites in a cohort of 627 patients with well-characterised NAFLD across the full spectrum of the disease.

## Materials and methods

### Study participants

Samples came from a cohort of 627 histologically characterised patients recruited from clinics at a number of leading international tertiary liver centres (UK, France, Germany, Brazil, and Italy) participating in the ‘European NAFLD Registry’ project (Clinicaltrials.gov NCT04442334). This cohort includes the full spectrum of disease, from histologically normal liver tissue through NAFL to NASH-F4 (cirrhosis). The protocol and detailed description of the Registry has recently been published.^15^ Briefly, patients with alternate diagnoses and aetiologies were excluded, including excessive alcohol intake (>30 g per day for males and >20 g for females), viral hepatitis, autoimmune liver diseases, and steatogenic medication use. Collection and use of samples and clinical data for this study was approved by the relevant local and/or national ethical review committee covering each participating centre, with all patients providing informed consent for participation. All participant recruitment and informed consent processes at recruitment centres were conducted in compliance with nationally accepted practice in the respective territory and in accordance with the World Medical Association Declaration of Helsinki 2018.

### Liver histology

Liver biopsy specimens (at least 1.6-cm length and ∼1-mm diameter) were formalin-fixed and paraffin-embedded. Tissue sections (3–4 μm thick) were stained with H&E and trichrome stain for visualising collagen. All cases were recruited at tertiary centres where liver biopsies were routinely assessed according to accepted criteria by experienced liver pathologists and scored using the well-validated National Institute of Digestive Diseases and Kidney NASH Clinical Research Network (NIDDK NASH-CRN) system.^16^ To ensure optimum data quality, biopsies were retrieved from archival storage where possible and scored centrally by a central expert liver pathologist, as previously described.^15^ Where archival samples were unavailable for central reading, the local liver pathologist's scores were used. All samples were scored according to the semiquantitative NASH-CRN NASH activity score (NAS) and the Fatty Liver Inhibition of Progression (FLIP) steatosis (S), activity (A), and fibrosis (F) (SAF) scoring system.^16^^,^^17^ Fibrosis was staged from F0 to F4 (cirrhosis).

### Metabolomics

Analyses of molecular lipids and polar metabolites are described in detail in the Supplementary information. In brief, for lipidomics, lipids from serum samples were extracted in randomised sample order using a modified version of the previously published Folch procedure, as applied recently,^18^ and analysed by ultrahigh-performance liquid chromatography (UHPLC) coupled to quadrupole-time-of-flight mass spectrometry (QTOFMS). MS data processing was performed using MZmine version 2.18.^19^ For the analysis of polar metabolites, serum samples were randomised, and sample preparation was carried out as described previously, using gas chromatography (GC) coupled to QTOFMS.^20^

### Statistical analysis

All analyses were carried out in the R statistical programming language environment (R Foundation for Statistical Computing, Vienna, Austria).^21^ Missing values in the lipidomic/polar metabolite data were replaced with imputed half-minimums for their respective feature, log_2_-transformed, and scaled to zero mean and unit variance. The sample intersection between the lipidomic, polar metabolomic, and clinical information datasets was retained. A detailed description of the statistical analysis methodology in provided in the Supplementary information.

## Results

### Study setting and data survey

Following integration of the metabolomics and clinical data, the study comprised 627 histologically characterised individuals across the full spectrum of NAFLD severity (Table 1), with a metabolomics dataset comprising 176 identified lipids and 36 quantified metabolites.

**Table 1:** **Demographic characteristics of the study population**.

Clinical features	N total (n=627)	Count/mean ± SD
Age (mean ± SD)	615	51.61 ± 12.68
Sex	626	
Male		339
Female		287
BMI (mean ± SD)	561	31.88 ± 6.32
T2DM	574	
No		311
Yes		263
HbA_1c_ (mmol/mol ± SD)		49.07 ± 25.09
ALT (mean ± SD)		64.87 ± 43.78
AST (mean ± SD)		45.2 ± 29.23
Platelet (×10^9^)		232.47 ± 69.40
Triglycerides (mmol/L)		3.06 ± 13.69
Total cholesterol (mmol/L)		5.42 ± 9.65
Steatosis grade	618	
0		36
1		210
2		247
3		125
Ballooning	618	
0		136
1		277
2		205
Kleiner lobular Inflammation	618	
0		88
1		347
2		163
3		20
Brunt fibrosis stage	627	
0		147
1		173
2		122
3		132
4		53
NAS (mean ± SD)	618	4.04 ± 1.71
NAS score ≥4		
No		226
Yes		392

ALT, alanine aminotransferase; AST, aspartate aminotransferase; HbA_1c_, haemoglobin A_1c_; NAS, NASH activity score; NASH, non-alcoholic steatohepatitis; T2DM, type 2 diabetes mellitus.

**Fig. 1:**
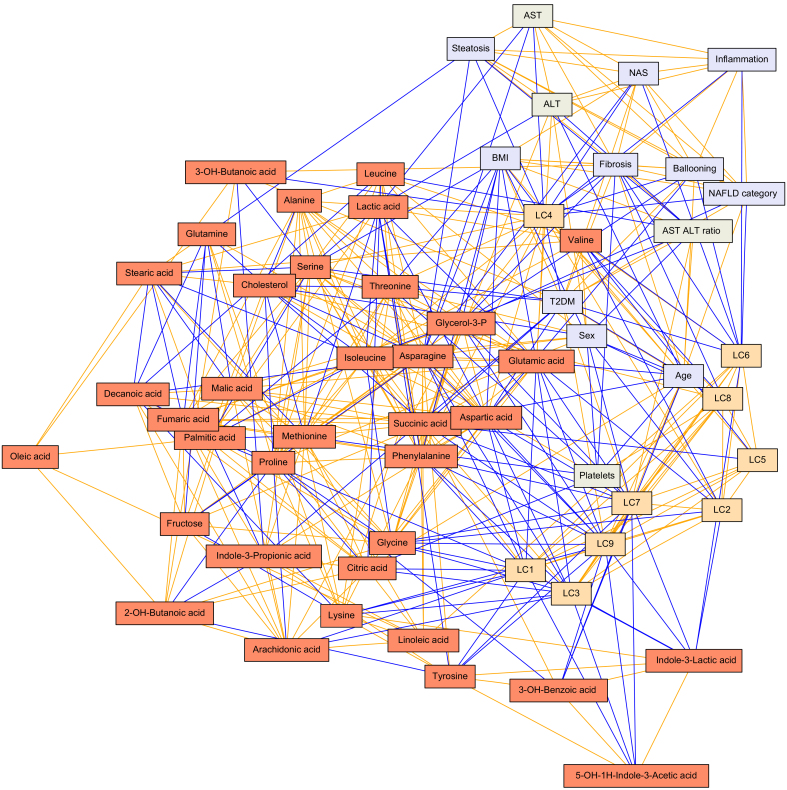
Putative partial correlation network. Associations between all study variables were filtered by the application of a NRR (<0.4; see the *Materials andMethods* section and Fig. S1B) to remove spurious associations. The remaining associations of interest are given as an interaction network, with edge thicknesses representing the strength of association, and the colours showing the direction of association (orange for positive association and blue for negative association). Nodes are collared purely by the dataset from which they originate, for clarity. Colours: LCs (orange), metabolites (red), clinical variables (blue), and blood-derived measures (grey). ALT, alanine aminotransferase; AST, aspartate aminotransferase; LC, lipid cluster; NAS, NASH activity score; NASH, non-alcoholic steatohepatitis; NRR, non-rejection rate; T2DM, type 2 diabetes mellitus.

To obtain a global overview of differences in metabolite levels across stages of NAFLD, we first applied model-based clustering to the metabolomics data. Clustering revealed 9 lipid clusters (LCs), in which clear groups of lipid classes are visible (Table 2). The polar metabolites data were found to not contain clusters, and as such was left unclustered as individual data features. Both lipidomics and polar metabolomics data were verified to not cluster by study site, as assessed by principal component analysis (PCA). Next, we studied the associations between all LCs, polar metabolites, and clinical variables (Fig. S1A). Based on the distribution of these pairwise associations (Fig. S1B), a conservative cut-off of non-rejection rates (NRRs) of <0.4 was chosen for retaining non-spurious associations. In addition, 858 correlations passed the NRR <0.4 threshold, therein rejecting 75% of associations as potentially spurious (Table S1). A partial correlation network was then constructed, connecting lipids, metabolites and clinical variables (Fig. 1). As this network is the culmination of analysing all possible associations between clinical variables, outcomes, lipids, and metabolites, with those associations likely to be spurious and pruned away, the network provides an at-a-glance crystallisation of the reliable associations between variables as well as the directionality of those associations in the data. For interpretation of relevance, the region of greatest interest is formed at the interface between node types, those being metabolites (red nodes), LCs (orange nodes), clinical variables (blue nodes), and blood measurements (grey nodes), as this is where metabolic links to outcomes and blood markers are suggested. For example, as seen in this network, lysophosphatidylcholines (LPCs; LC4) were found to be central to a cluster of features comprising 9 important clinical variables describing both grade and stage of NAFLD, including degree of steatosis (*r* = -0.13), NAFLD grade (NAFL *vs*. NASH; *r* = -0.14; LPCs being lower in NASH) and fibrosis stage (*r* = -0.19). Likewise, the ether PCs (PC(O)s; LC6) connected to 6 clinical variables, including to fibrosis (*r* = -0.12) and NASH (*r* = -0.12). Additionally, 2 further metabolites were linked directly with fibrosis: 3-OH-butanoic acid (*r* = 0.20) and glycerol-3-phosphate (*r* = -0.10). Whereas 3-OH-butanoic acid also linked with diagnosis of T2DM (r = 0.21), glycerol-3-phosphate did not attain such a link in this analysis. Conversely, glycerol-3-phosphate weakly associated with ballooning of cells in the liver (r = -0.03), whereas 3-OH butanoic acid did not. These interactions are also depicted in the correlation plot (Fig. S1A, where, as opposed to focusing on showing interconnectedness, the correlation plot focuses on showing strengths of associations.

**Table 2:** **Description of LCs**.

Cluster	Main lipid classes represented	Examples
1	CEs + TGs	TG(50:0),CE(18:0),TG(18:1/18:1/16/0),TG(54:4)
2	PCs (high carbon #), Cers	PC(40:5), Cer(d18:1/23:0), Cer(d18:1/23:0)
3	TGs	TG(14:0/16:0/18:1),TG(49:0),TG(56:2), TG(45:0)
4	LPCs	LPC(16:0e), LPC(18:1), LPC(22:6),LPC(20:3)
5	PCs + 1 PE	PC(38:6), PC(18:0p/22:6), PC(40:8), PE(P-10:0/22:6)
6	PC(O)s	PC(O-32:0), PC(O-40:6), PC(O-38:5), PC(O-36:3)
7	PEs	PE(16:0/18:1), PE(34:2), PE(38:4), PE(38:6)
8	SMs	SM(d32:1), SM(d42:2), SM(d36:0), SM(d18:1/24:0)
9	TGs (lowest and highest C #)	TG(14:0/18:2/18:2), TG(18:2/22:5/16:0), TG(58:9)

CE, cholesterol ester; Cer, ceramide; LC, lipid cluster; LPC, lysophosphatidylcholine; PC, phosphatidylcholine; PC(O), ether PC; PE, phosphatidylethanolamine; SM, sphingomyelin; TG, triacylglycerol.

### Metabolites associate with increasing histological disease severity

**Fig. 2:**
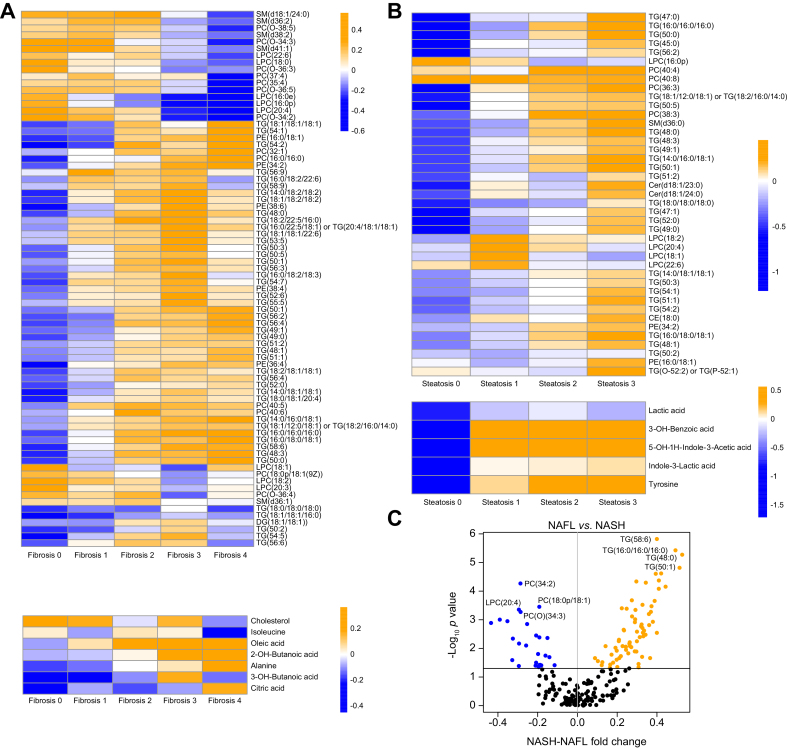
Lipid and polar metabolites associated with NAFLD fibrosis. Heat maps of features changing significantly (*p* <0.05 in ANOVA and/or Tukey HSD analyses) across (A) Kleiner fibrosis scores and (B) Kleiner steatosis scores. Each coloured cell represents the median value of a given feature across all samples in that fibrosis group. Colour bars denote magnitude of change in the level of that lipid/polar metabolite, with orange/blue depicting relatively higher/lower levels (within feature). Rows are clustered for clarity (dendrogram removed for clarity). All cells in the heat map represent the median value of a given lipid/polar metabolite for all individuals in that fibrosis (A) or steatosis (B) group. (C) A volcano plot depicting the median fold changes (*x*-axis) occurring in both lipids and metabolites between clinically rated NAFL *vs.* NASH, those in orange/blue having significantly increased/decreased in NASH. The 4 greatest significances for both increases and decreases in the levels are listed as examples. CE, cholesterol ester; Cer, ceramide; HSD, honest significant difference; LPC, lysophosphatidylcholine; NAFL, non-alcoholic fatty liver; NAFLD, non-alcoholic fatty liver disease; NASH, non-alcoholic steatohepatitis; PC, phosphatidylcholine; PE, phosphatidylethanolamine; SM, sphingomyelin; TG, triacylglycerol.

An overview of metabolites changes across the NAFLD spectrum (histological grade and stage) is summarised in Fig. 2. Feature-clustered heat maps showing stepwise changes in metabolite profiles with increasing degree of steatosis and stage of fibrosis are shown (Fig. 2A and B) for all lipids/polar metabolites achieving significance (*p* <0.05) in either an ANOVA or Tukey honest significant difference (HSD) analysis. It should be noted that, as log_2_-transformed and autoscaled data are used throughout all heat maps and intercomparisons for calculation of changes, the calculated changes function as fold change on a log_2_ scale, as opposed to any direct changes in units of concentration. For the grade of disease activity using the binary categories of NAFL *vs.* NASH, as there are only 2 disease states, results are represented as a volcano plot (Fig. 2C). The full lists of all autoscaled log_2_ changes for all lipids and metabolites, as well as significance values for all ANOVA/Tukey HSD results, are provided in Table S2 under the relevant spreadsheet tabs for fibrosis, steatosis, and NASH. Further, the trend of direction of change of all lipids/metabolites identified as changing significantly from any of the 3 perspectives (steatosis, steatohepatitis, and fibrosis) are summarised as a heat map in Fig. S2.

**Fig. 3:**
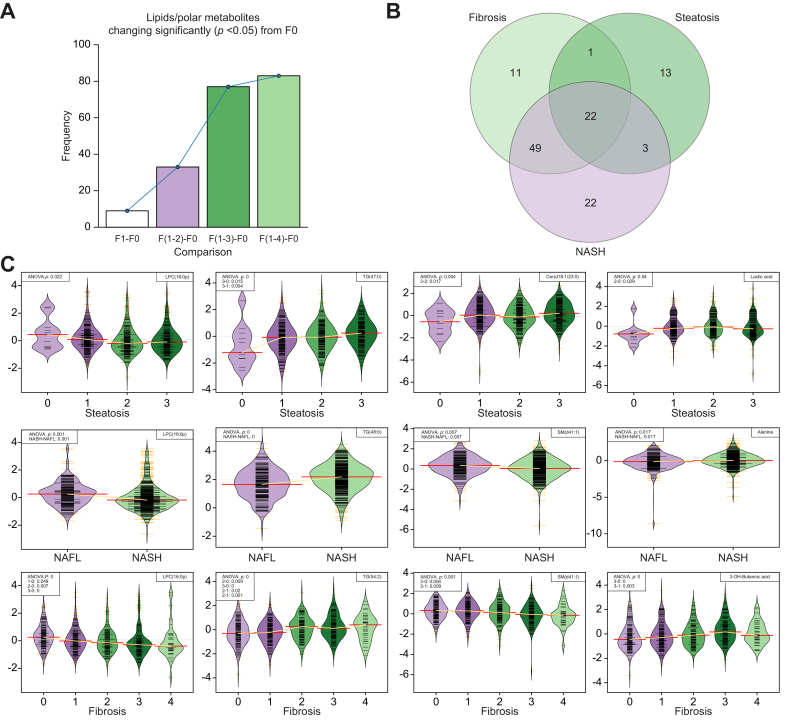
Representative examples of lipid and metabolite changes across fibrosis and steatosis and between NAFL/NASH states. (A) Number of changing metabolites across Kleiner fibrosis stages in all (n = 627) participants, showing the number of metabolites changing between each stage (F1–F4) as compared with F0. (B) Venn diagram depicting which metabolites significantly change across fibrosis stages (light green) and steatosis grades (green) or between NAFL/NASH diagnoses (purple), revealing the existence of both overlapping and, crucially, specific metabolic signatures of 3 clinical perspectives of NAFLD. (C) Levels of lipids/metabolites across the 5 stages of fibrosis as per the Kleiner scoring system (bottom row), the 4 steatosis scores (top row), and between NAFL and NASH (middle row). For each row, 3 representative lipids and 1 metabolite are given. ANOVA and Tukey HSD *p* values shown in the upper left of each panel only given if they satisfy significance threshold of (*p* <0.05). Cer, ceramide; HSD, honest significant difference; LPC, lysophosphatidylcholine; NAFL, non-alcoholic fatty liver; NAFLD, non-alcoholic fatty liver disease; NASH, non-alcoholic steatohepatitis; SM, sphingomyelin; TG, triacylglycerol.

Observing the cumulative number of changing lipids and polar metabolites across fibrosis progression, using F0 as a reference point, a total of 9 metabolites changed by F1, 33 by F2, 77 by F3, and 83 by F4 (Fig. 3A), thereby showing additions of 9, 24, 44, and 6 features, respectively, changing significantly at each stage of fibrosis progression. It must be noted that only the first significant change across F0–F4 was counted for each lipid or metabolite; that is, a given lipid/polar metabolite was counted as having changed significantly only at the earliest stage of fibrosis in which it changed, to avoid erroneously inflating the number of changing lipids/metabolites at later time points.

Lipids and polar metabolites with significantly (*p* <0.05) differing levels, by ANOVA or Tukey HSD, between participants across steatosis, NAFL/NASH grade, and fibrosis stage were tabulated (Table S2). From this table (Table S2) is derived a Venn diagram (Fig. 3B) showing the distribution of lipids and metabolites unique to and common between these 3 key histological features of NAFLD severity. Given that steatohepatitic and fibrotic processes frequently coexist in the same biopsies, mention is given here also to those lipids/metabolites common to fibrosis and NAFL *vs.* NASH. The full lists of all autoscaled log_2_ changes for all lipids and metabolites, as well as significance values for all ANOVA/Tukey HSD results, are provided in Table S2 under the relevant spreadsheet tabs for fibrosis, steatosis, and NASH.

For visualisation at an individual level, a selection of representative lipids and polar metabolites found to change significantly (ANOVA *p* <0.05) across (1) fibrosis stages and (2) steatosis grades, and (3) between NAFL *vs*. NASH are given in Fig. 3C.

#### Steatosis

In total, 9 lipids and 6 polar metabolites changed uniquely across steatosis grades but did not alter with either disease activity or fibrosis stage. These included 2 TGs, 4 PCs, 2 Cers, and 1 cholesterol ester (CE). There was little overlap between steatosis and NAFL/NASH, while excluding fibrosis, adding only 4 TGs to the signature, for a total of 6 TGs.

#### NAFL *vs.* NASH

Differentiating only between NAFL and NASH, and excluding changes across steatosis and fibrosis, a signature of (n = 18) lipids was found, including 9 TGs (increased in NASH), 2 sphingomyelins (SMs), 6 PC(O)s, and 1 phosphatidylethanolamine (PE; decreased in NASH).

#### Fibrosis

In total, 8 lipids and 7 polar metabolites were found to change solely with fibrosis stage, namely PC(32:1), PC(35:4), PC(37:4), PC(40:5), PC(40:6), SM(d36:1), SM(d36:2), and SM(d38:2), and the polar metabolites were 2-hydroxybutanoic acid (2-HB), 3-hydroxybutanoic acid (3-HB), citric acid, isoleucine, lysine, and oleic acid.

#### Fibrosis and NASH

Consistent with the frequent co-existence of steatohepatitic and fibrotic processes as disease evolves, a considerable overlap between associated metabolites was observed, with 46 lipids and 1 other metabolite (alanine) identified. The lipids were 29 TGs, 8 PCs, 3 LPCs, 3 PEs, 2 SMs, and 1 DG.

#### Lipid/metabolite profiles common between steatosis, fibrosis, and NASH

At the core of all 3 histological disease components, 27 lipids, but no polar metabolites, were found to change with increasing disease severity. This group of lipids is dominated by 20 TGs, followed by 5 LPCs and 2 PEs.

### Overlapping and sexually dimorphic profiles of lipids and metabolites across NAFLD perspectives

In total, 3 Venn diagrams (Fig. S3A) show the overlaps of lipids and metabolites significantly changing in the same direction across the severity scores of the 3 selected perspectives of fibrosis, steatosis, and NASH when using only male participants (n = 339, 54%), only female participants (n = 228, 45%), or both sexes together. Further, similarly to that in Fig. 3A, the total number of lipids/metabolites whose levels significantly differ from F0 are given in bar plot in Fig. S3B. As before in Fig. 3A, this is represented as pseudo-longitudinal as being ‘from F0 to F4 severity’, where a lipid/metabolite is only counted as having changed significantly once, at the lowest fibrosis score where it changes significantly, and not again at higher fibrosis scores. A summary table providing the changes of all direction of lipids/metabolites and their significances across steatosis, fibrosis, and NAFL/NASH in all 3 (male, female, and both) analyses is given in Table S3.

### Identification of discriminative molecular signatures for stratification of NAFLD fibrosis

To further characterise the metabolic evolution of NAFLD, 2 well-described disease outcome deflection points were studied, comparing mild disease with either ‘clinically significant’ fibrosis (NASH F2–F4) or ‘advanced’ fibrosis (NASH F3–F4),^22^ by using discriminative modelling.

**Fig. 4:**
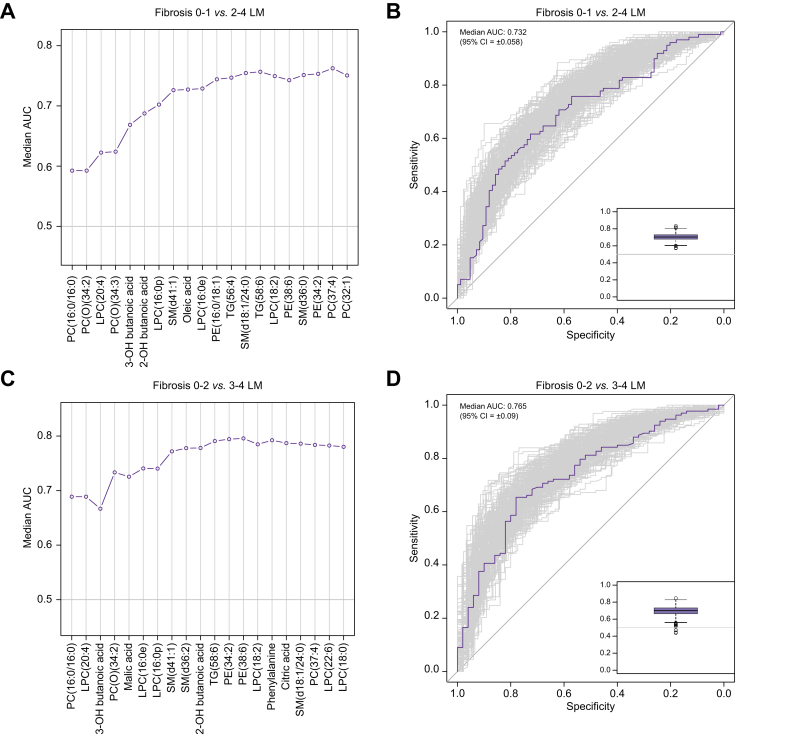
Lipids and metabolites’ classification of patients with NAFLD before and after the metabolic tipping point of NAFLD. Results of random forest predicting (A and B) NAFLD Kleiner fibrosis score (0–1) *vs*. (2–4), and (C and D) Kleiner fibrosis score (0–2) *vs.* (3–4). (A) and (C) show the progress of recursive feature addition, recursively adding the most important features, plotting median AUC of the model as more features are added. (B) and (D) show the ROC curves (main panel) and distribution of AUCs (inset box plot) when using the minimal feature sets (n = 2,001 iterations). LM, lipid and metabolite; LPC, lysophosphatidylcholine; NAFLD, non-alcoholic fatty liver disease; PC, phosphatidylcholine; PE, phosphatidylethanolamine; SM, sphingomyelin; TG, triacylglycerol.

The goal of this study was not to develop biomarkers but to examine pathophysiological changes as the disease evolves. However, as an exemplar of how the metabolomic signatures may be leveraged, median AUCs during recursive feature addition to generate minimal feature sets for both discrimination tasks are shown for the lipid and metabolite (LM) analyses in Fig. 4A and C, with receiving operator characteristic (ROC) curves when using these feature sets shown in Fig. 4B and D. Similar plots are given for all 6 classification tasks in Figs. S4 and S5.

Discriminative models using lipids and polar metabolites only (LM) achieved median AUCs of 0.732 (95% CI 0.058; using 12 features) and 0.765 (95% CI 0.09; using 13 features) for the F0–F1 *vs*. F2–F4 task and F0–F2 *vs*. F3–F4 tasks, respectively. These compared favourably with models built using standard clinical data points used in clinical practice (C; n = 8 features; BMI, liver aspartate aminotransferase [AST], liver alanine aminotransferase [ALT], liver AST/ALT ratio, age, sex, presence of T2DM, and platelet count), which achieved median AUCs of 0.746 (95% CI 0.056) and 0.778 (95% CI 0.051). Combining clinical features to the lipids and polar metabolites (LMC) produced minor improvement in median AUC (+0.004 and +0.022), showing competitive performance of lipids and polar metabolites with the top clinical features. The relative performances of the models built with all input predictor sets (C, LM, and LMC) for both classification tasks (total of n = 6 performances) using our minimal feature sets are given and contrasted in Table 3.

**Table 3:** **Summary of classification task performances**.

Dataset	Fibrosis 0–1 *vs.* 2–4		Fibrosis 0–2 *vs*. 3–4	
	Median AUC (95% CI)	Minimal features (n)	Median AUC (95% CI)	Minimal features (n)
LM	0.73 (0.058)	13	0.77 (0.053)	12
LMC	0.77 (0.048)	9	0.78 (0.044)	10
C	0.75 (0.056)	7	0.78 (0.051)	7
Improvement by adding (LM) to (C)	0.022		0.004	
Performance of (LM alone) as % of (C)	98.1		98.3	

C, clinical variables used as predictors; LM, lipids and metabolites used as predictors; LMC, lipids, metabolites, and clinical variables used as predictors.

## Discussion

Our results provide a detailed overview of lipids, polar metabolites, and key clinical variables and observations in NAFLD, providing potential insights into the metabolic basis of pathophysiology and disease natural history. As a key novel finding, our analysis pinpoints a critical transition point in the metabolic profiles of patients with NAFLD, concurrent with the progression from fibrosis stage F2–F3. Interestingly, this increase in fibrosis stage is also the stage at which both all-cause and liver-related mortality begin to markedly increase^22^ and underscores the relevance of this work as a metabolic snapshot across the F2–F3 transition as well as identifying putative discriminant lipids/metabolites. Here, the largest number (n = 43) of significant changes in metabolite levels occurred between stages F2 and F3. Conversely, a relatively low number (n = 4) of lipids/polar metabolites specifically changed at F4, suggesting that the liver already exhibits a metabolically ‘poised’ state before progression to cirrhosis, with hardly any change in its metabolic signature at compensated F4 despite a large change in fibrosis burden. This underscores the importance of the F2–F3 transition and thus the critical importance of preventing it. However, it must be made clear that the cohort, and consequently this study, is of a cross-sectional nature, rather than a longitudinal nature. Therefore, although we refer to changes in lipid and metabolite levels across the spectrum of disease, these should be seen as commonly and robustly observed profiles of levels of lipids and metabolites, corresponding to disease severity, given the considerable size of the cohort. However, these cannot be said to be observed changes in lipid and metabolite levels within individual patients at different time points.

We identify a set of circulating TGs, PCs, Cers, and 1 CE that appear to pertain solely to hepatic steatosis burden, and not to NASH or fibrosis progression. Given the hepatotoxicity of Cers and the increasing understanding of their role in cellular damage in NAFLD,^9^^,^^23^ it is of interest that the Cers identified in our analysis were unique to steatosis and did not significantly change from the perspective of NAFL to NASH conversion or fibrosis. Interestingly, SMs are implicated as the lipids that are converted to hepatotoxic Cers, and yet SMs did not form part of the steatosis-unique metabolic signature (although SM(d36:0) was shared as an overlap between fibrosis and steatosis signatures). Also unique to steatosis were metabolites of microbial origin such as 3-OH-benzoic acid, 5-OH-1H-indole-3-acetic acid, and indole-3-lactic acid, all of which were found to be raised as steatosis progresses, thus highlighting the potential role of gut microbiome in steatosis.^24^

Despite the expected overlap in metabolic signature between NAFL to NASH transition and fibrosis progression, there remained 18 lipids associated solely with the presence of NASH, independent of fibrosis stage. We identify a signature of increases in 9 TGs and decreases in 6 PCs, 2 SMs, and 1 PE, which are specific to NAFL–NASH conversion. It must be noted that the presence of an NAFL to NASH conversion-unique signature, apparently distinct from the signature of the progression of fibrosis may be caused by potentially increased statistical power of the ANOVA/Tukey HSD tests on lipids and metabolites between NAFL and NASH, given that these groups contained larger numbers of samples, not being spread across the 5 stages of fibrosis (F0–F4). Regardless, this metabolic signature may be capturing clinically informative metabolic changes that warn of NAFL to NASH progression but that occur independent of steatosis or fibrosis. The TGs that increase in NASH include those TGs with low carbon number and double bond count, which were previously found to be associated with *de novo* lipogenesis,^25^ insulin resistance,^26^ NAFLD,^10^ and with increased risk of type 2 diabetes.^27^

As compared with other assessments performed at biopsy (including steatosis, ballooning, and inflammation), fibrosis stage is considered the most tractable surrogate marker of likely clinical outcome in NAFLD.^22^ We demonstrate here the existence of a fibrosis-specific metabolic metabolomic/lipidomic signature. This is remarkable, particularly considering the expected coexistence of the steatohepatitic and fibrotic processes as the disease evolves. There were 8 lipids and 7 polar metabolites unique to fibrosis progression, to the exclusion of both steatosis progression and NAFL–NASH conversion. Here, we detect increases in the levels of 3 PCs, along with oleic acid, lysine, citric acid, 3-HB, and 2-HB. Conversely, we detect decreases in the levels of 3 SMs and 2 other PCs, along with isoleucine and cholesterol. The fact that a metabolic signature unique to fibrosis was identified as distinct from that of NAFL–NASH progression supports the idea that these facets of NAFLD, although undoubtedly linked, still contain distinct and disease-process-specific information with clinical utility for stratification/diagnosis/guiding intervention.

In this work, we identified a clear metabolic tipping point at the F2–F3 transition in this cohort of patients with NAFLD. We also demonstrate a fibrosis-specific metabolic signature and a steatohepatitis signature, albeit with some overlap between these.

NAFLD molecular signatures are known to differ between patients in a sexually dimorphic manner.^28^^,^^29^ Our data were thus also split into male and female and re-analysed using the same analysis pipeline to reveal both overlap and disparity in significantly changing lipids and metabolites across severity scores for fibrosis, steatosis, and NASH (Fig. S3A). Although lipids/metabolites mostly showed altered levels in the same direction in both sexes, some sex-specific changes were also identified. In fibrosis, 5 lipids/metabolites showed significantly different levels across severity scores only in males and 17 likewise only in females (Fig. S3A). Discounting the ‘both sexes’ analyses, and assuming only male *vs.* female assignment of all lipids/metabolites, would give 44 (45%) male-unique, 20 (21%) shared, and 33 (34%) female-unique in fibrosis, 15 (25%) male-unique, 18 (31%) shared, and 26 (44%) female-unique in steatosis, with 82 (68%) male-unique, 16 (22%) shared, and 12 (10%) female-unique in NAFL/NASH. Despite these clear sex differences, the ‘both sexes’ analyses identified 75 of 97 (77%) of those lipids/metabolites agreed upon by the separate male and female analyses in the case of fibrosis, 35 of 59 (59%) for steatosis and 92 of 120 (77%) for NAFL/NASH. The full list of lipid/metabolite direction changes and significances for fibrosis, steatosis, and NAFL/NASH are given for all 3 (male, female, and both) analyses in Table S3. In the case of fibrosis, the changes observed between F2 and F3 may occur slightly earlier in females (Fig. S3B), whereas for ‘both sexes’ and for males, the more pronounced, typical jump across the F2–F3 point occurs. When we applied machine learning techniques to all participants, for discrimination between less and more severe NAFLD, classification was best across the aforementioned F2–F3 transition. Here, and to our surprise, sex was the most unimportant feature when classifying fibrosis, in all models constructed, where it ranked at the lowest position of importance. This was true even when using only clinical variables to distinguish F0–F2 *vs*. F3–F4 (see Table S4).

Across the F2–F3 transition, using the whole dataset (male and female), we observed increases in several PCs and TGs and decreases in PC(O)s, LPCs, and SMs, as well as several metabolites including 2- and 3-HB. Increased 2-HB was previously found as an early indicator of insulin resistance^30^ and worsening glucose tolerance.^31^ In addition, 2-HB is produced in the liver as a by-product of increased hepatic glutathione biosynthesis, which is induced by metabolic stress such as oxidative stress or increased detoxification demands. In line with this, ether lipids such as those found decreased between F2 and F3 in our study, are considered as endogenous antioxidants, while also having several other biological functions.^32^ As the ether lipids require peroxisomes for their synthesis, they are mainly produced in tissues and organs where peroxisomes are abundant, such as in the liver. The ether lipids were previously found associated with obesity and type 2 diabetes.[33], [34], [35] In addition, a decrease of specific LPCs has been associated with pre-diabetes, obesity, and type 2 diabetes,^34^^,^^36^^,^^37^ although the underlying mechanisms linking these lipids with the disease progression are poorly understood. Moreover, SMs, which are major components of low-density lipoprotein (LDL) particles, were previously found associated with increased risk of type 2 diabetes,^35^^,^^37^ although the association appears to be BMI dependent.^33^^,^^38^

### Limitations

We acknowledge that the detailed identification of the underlying mechanisms driving the metabolic transition observed at F2–F3 awaits further study. Knowledge of these metabolites’ changes and the underlying metabolic pathways may also help in further refining the staging of NAFLD as well as provide targets for future pharmacological interventions. Furthermore, although our study suggests that lipids/metabolites may be useful in the discrimination of patients before, or across, this metabolic ‘tipping point’ (F2–F3), establishing biomarker performance was outside the scope of this study, and further prospective studies will be needed to assess and validate the potential diagnostic value of the metabolites identified. This is currently being assessed within the EU Innovative Medicines Initiative (IMI2) Liver Investigation: Testing Marker Utility in Steatohepatitis (LITMUS) project.^8^ Finally, we acknowledge that our investigation is a ‘within-disease’ study. Once normal ranges for different classes of molecular lipids and metabolites are defined in healthy individuals, which is an ongoing effort within the lipidomics community,^8^^,^^39^ it will be important to establish how those levels compare with those at different stages of NAFLD.

### Conclusions

Taken together, our findings, from an initial top-down approach, provide a comprehensive overview of the metabolic network across the full spectrum of NAFLD, integrated with clinical measures and assessments. We go on to provide high-resolution metabolomic interrogation of NAFLD, both as a whole and then, uniquely, from 3 different crucial perspectives of the disease, namely, steatosis, NAFL–NASH conversion, and fibrosis, identifying metabolic signatures unique and common between each, even in light of the sexually dimorphic nature of the disease. We additionally observe a potentially important metabolic tipping point at the F2–F3 transition, where our data support the role of metabolic stress and specifically oxidative stress. Notably, F2–F3 progression associates with several metabolic markers previously associated with development of type 2 diabetes. Finally, we demonstrate utility of our lipidomic and polar metabolomic datasets in comparison with established clinical measures in discriminating between patients with NAFLD before or after this metabolic tipping point, suggesting a potential use in clinical practice.

## Financial support

This study received support from the Elucidating Pathways of Steatohepatitis (EPoS) consortium funded by the 10.13039/100010661Horizon 2020 Framework Program of the European Union under Grant Agreement 634413; the Liver Investigation: Testing Marker Utility in Steatohepatitis (LITMUS) consortium funded by the 10.13039/501100010767Innovative Medicines Initiative (IMI2) Program of the European Union under Grant Agreement 777377; this Joint Undertaking received support from the 10.13039/100010661European Union’s Horizon 2020 Research and Innovation Programme and 10.13039/100013322EFPIA; and the 10.13039/501100012295Newcastle NIHR Biomedical Research Centre and the European NAFLD Registry.

## Authors’ contributions

Proposed and designed the study: MO, QMA. Supervised the study: MO, QMA, AKD, TH. Performed data analysis: AM. Performed metabolomics analysis: DG. Supervised metabolomics analysis: TH. Contributed to clinical data acquisition in the European NAFLD Registry cohort: VR, MA, JB, SP, CO, EB, JMS, QMA. Coordinated clinical data acquisition in the European NAFLD Registry cohort: QMA. Wrote the manuscript: AM, MO. Provided further major contributions to the manuscript: QMA, AKD, OG, TH. Critically revised the manuscript for intellectual content and approved the final manuscript: All authors.The guarantor of this work and, as such, had full access to all of the data in the study and takes responsibility for the integrity of the data and the accuracy of the data analysis: MO.

## Data availability statement

Data from this study are available from the Metabolomics Workbench (https://www.metabolomicsworkbench.org/) at DOI https://doi.org/10.21228/M85976.

## Conflicts of interest

QMA is Coordinator of the EU IMI-2 LITMUS consortium, which is funded by the EU Horizon 2020 programme and EFPIA. He reports research grant funding: Allergan/Tobira, AstraZeneca, GlaxoSmithKline, Glympse Bio, Novartis Pharma AG, and Pfizer Ltd. Consultancy: 89Bio, Allergan/Tobira, Altimmune, AstraZeneca, Axcella, Blade, BMS, BNN Cardio, Cirius, CymaBay, EcoR1, E3Bio, Eli Lilly & Company Ltd., Galmed, Genentech, Genfit SA, Gilead, Grunthal, HistoIndex, Indalo, Intercept Pharma Europe Ltd., Inventiva, IQVIA, Janssen, Madrigal, MedImmune, Medpace, Metacrine, NGMBio, North Sea Therapeutics, Novartis, Novo Nordisk A/S, PathAI, Pfizer Ltd., Poxel, ProSciento, Raptor Pharma, Roche, Servier, Terns, The Medicines Company, and Viking Therapeutics. Speaker: Abbott Laboratories, Allergan/Tobira, BMS, Clinical Care Options, Falk, Fishawack, Genfit SA, Gilead, Integritas Communications, Kenes, and Medscape. Royalties: Elsevier Ltd.

Please refer to the accompanying ICMJE disclosure forms for further details.
