# Supplementary material for: Metabolic signatures across the full spectrum of non-alcoholic fatty liver disease
Source: JHEP Rep. 2022 Mar 26;4(5):100477. doi: 10.1016/j.jhepr.2022.100477 (PMC9006858; doi:10.1016/j.jhepr.2022.100477)
Supplement: Multimedia component 5 [file mmc5.pdf]

Metabolic signatures across the full spectrum of non-alcoholic fatty liver disease

Aidan McGlinchey, Dawei Geng, Olivier Govaere, Vlad Ratziu, Michael Allison, Jerome Bousier, Salvatore Petta, Claudia P Oliveira, Elisabetta Bugianesi, Jorn Schattenberg, Ann Daly, Tuulia Hyötyläinen, Quentin M. Anstee, Matej Orešič

Table of contents

Supplementary methods 2

Supplementary figures 9

Supplementary tables 14

Supplementary references 16

Supplementary methods

Analysis of molecular lipids (lipidomics)

Serum samples were randomized and extracted using a modified version of the previously-published Folch procedure, as applied recently [1]. In short, 10 µL of 0.9% NaCl and, 120 µL of CHCl3: MeOH (2:1, v/v) containing the internal standards (c = 2.5 µg/mL) was added to 10 µL of each serum sample. The standard solution contained the following compounds: 1,2-diheptadecanoyl-sn-glycero-3-phosphoethanolamine (PE(17:0/17:0)), N-heptadecanoyl-D-erythro-sphingosylphosphorylcholine (SM(d18:1/17:0)), N-heptadecanoyl-D-erythro-sphingosine (Cer(d18:1/17:0)), 1,2-diheptadecanoyl-sn-glycero-3-phosphocholine (PC(17:0/17:0)), 1-heptadecanoyl-2-hydroxy-sn-glycero-3-phosphocholine (LPC(17:0)) and 1-palmitoyl-d31-2-oleoyl-sn-glycero-3-phosphocholine (PC(16:0/d31/18:1)), were purchased from Avanti Polar Lipids, Inc. (Alabaster, AL, USA), and, triheptadecanoylglycerol (TG(17:0/17:0/17:0)) was purchased from Larodan AB (Solna, Sweden). The samples were vortex mixed and incubated on ice for 30 min after which they were centrifuged (9400 × g, 3 min). 60 µL from the lower layer of each sample was then transferred to a glass vial with an insert and 60 µL of CHCl3: MeOH (2:1, v/v) was added to each sample. The samples were stored at -80 °C until analysis.

Calibration curves using 1-hexadecyl-2-(9Z-octadecenoyl)-sn-glycero-3-phosphocholine (PC(16:0e/18:1(9Z))), 1-(1Z-octadecenyl)-2-(9Z-octadecenoyl)-sn-glycero-3-phosphocholine (PC(18:0p/18:1(9Z))), 1-stearoyl-2-hydroxy-sn-glycero-3-phosphocholine (LPC(18:0)), 1-oleoyl-2-hydroxy-sn-glycero-3-phosphocholine (LPC(18:1)), 1-palmitoyl-2-oleoyl-sn-glycero-3-phosphoethanolamine (PE(16:0/18:1)), 1,2-Dioctadecanoyl- -sn-glycero-3-phosphocholine (PC(18:0/18:0)), 1-Hexadecanoyl-2-oleoyl-sn-glycero-3-phosphocholine (PC(16:0/18:1) and 1-stearoyl-2-linoleoyl-sn-glycerol (DG(18:0/18:2)), 1-(9Z-octadecenoyl)-sn-glycero-3-phosphoethanolamine (LPE(18:1)), N-(9Z-octadecenoyl)-sphinganine (Cer(d18:0/18:1(9Z))), 1-

hexadecyl-2-(9Z-octadecenoyl)-sn-glycero-3-phosphoethanolamine (PE(16:0/18:1)) from Avanti Polar Lipids, 1-Palmitoyl-2-Hydroxy-sn-Glycero-3-Phosphatidylcholine (LPC(16:0)), 1,2,3 trihexadecanoalglycerol (TG(16:0/16:0/16:0)), 1,2,3-trioctadecanoylglycerol (TG(18:0/18:0/18:0)) and 3β-hydroxy-5-cholestene-3-stearate (ChoE(18:0)), 3β-Hydroxy-5-cholestene-3-linoleate (ChoE(18:2)) from Larodan, were prepared to the following concentration levels: 100, 500, 1000, 1500, 2000 and 2500 ng/mL (in CHCl3:MeOH, 2:1, v/v) including 1250 ng/mL of each internal standard.

The samples were analyzed by ultra-high-performance liquid chromatography quadrupole time-of-flight mass spectrometry (UHPLC-QTOFMS). Briefly, the UHPLC system used in this work was a 1290 Infinity II system from Agilent Technologies (Santa Clara, CA, USA). The system was equipped with a multi sampler (maintained at 10 °C), a quaternary solvent manager and a column thermostat (maintained at 50 °C). Injection volume was 1 µL and the separations were performed on an ACQUITY UPLC® BEH C18 column (2.1 mm × 100 mm, particle size 1.7 µm) by Waters (Milford, MA, USA). The mass spectrometer coupled to the UHPLC was a 6545 QTOF from Agilent Technologies interfaced with a dual jet stream electrospray (Ddual ESI) ion source. All analyses were performed in positive ion mode and MassHunter B.06.01 (Agilent Technologies) was used for all data acquisition. Quality control was performed throughout the dataset by including blanks, pure standard samples, extracted standard samples and control serum samples, including in-house serum and a pooled QC with an aliquot of each sample was collected and pooled and used as quality control sample.

Relative standard deviations (% RSDs) for identified lipids in the control serum samples (n = 13) and in the pooled serum samples (n = 54) were on average 22.4% and 17.5%, respectively.

Mass spectrometry data processing was performed using the open source software package MZmine 2.18 [2]. The following steps were applied in this processing: (i) Crop filtering with a

m/z range of 350 – 1200 m/z and an RT range of 2.0 to 12 minutes, (ii) Mass detection with a noise level of 750, (iii) Chromatogram builder with a minimum time span of 0.08 min, minimum height of 1000 and a m/z tolerance of 0.006 m/z or 10.0 ppm, (iv) Chromatogram deconvolution using the local minimum search algorithm with a 70% chromatographic threshold, 0.05 min minimum RT range, 5% minimum relative height, 1200 minimum absolute height, a minimum ration of peak top/edge of 1.2 and a peak duration range of 0.08 - 5.0, (v), Isotopic peak grouper with a m/z tolerance of 5.0 ppm, RT tolerance of 0.05 min, maximum charge of 2 and with the most intense isotope set as the representative isotope, (vi) Peak filter with minimum 12 data points, a FWHM between 0.0 and 0.2, tailing factor between 0.45 and 2.22 and asymmetry factor between 0.40 and 2.50, (vii) Join aligner with a m/z tolerance of 0.009 or 10.0 ppm and a weight for of 2, a RT tolerance of 0.1 min and a weight of 1 and with no requirement of charge state or ID and no comparison of isotope pattern, (viii) Peak list row filter with a minimum of 10% of the samples (ix) Gap filling using the same RT and m/z range gap filler algorithm with an m/z tolerance of 0.009 m/z or 11.0 ppm, (x) Identification of lipids using a custom database search with an m/z tolerance of 0.009 m/z or 10.0 ppm and a RT tolerance of 0.1 min, and (xi) Normalization using internal standards PE(17:0/17:0), SM(d18:1/17:0), Cer(d18:1/17:0), LPC(17:0), TG(17:0/17:0/17:0) and PC(16:0/d30/18:1)) for identified lipids and closest ISTD for the unknown lipids followed by calculation of the concentrations based on lipid-class concentration curves.

Analysis of polar metabolites

Serum samples were randomized and sample preparation was carried out as described previously [3]. In short, 400 µL of MeOH containing ISTDs (heptadecanoic acid, deuterium-labeled DL-valine, deuterium-labeled succinic acid, and deuterium-labeled glutamic acid, c = 1 µg/mL) was added to 30 µl of the serum samples which were vortex mixed and incubated on ice for 30 min after which they were centrifuged (9400 × g, 3 min) and 350 µL of the supernatant was collected

after centrifugation. The solvent was evaporated to dryness and 25 µL of MOX reagent was added and the sample was incubated for 60 min at 45 °C. 25 µL of MSTFA was added and after 60 min incubation at 45 °C 25 µL of the retention index standard mixture (n-alkanes, c=10 µg/mL) was added.

The analyses were carried out on an Agilent 7890B GC coupled to 7200 QTOF MS. Injection volume was 1 µL with 100:1 cold solvent split on PTV at 70 °C, heating to 300 °C at 120 °C/minute. Column: Zebron ZB-SemiVolatiles. Length: 20m, I.D. 0.18mm, film thickness: 0.18 µm. With initial Helium flow 1.2 mL/min, increasing to 2.4 mL/min after 16 mins. Oven temperature program: 50 °C (5 min), then to 270°C at 20 °C/min and then to 300 °C at 40 °C/min (5 min). EI source: 250 °C, 70 eV electron energy, 35µA emission, solvent delay 3 min. Mass range 55 to 650 amu, acquisition rate 5 spectra/s, acquisition time 200 ms/spectrum. Quad at 150 °C, 1.5 mL/min N2 collision flow, aux-2 temperature: 280 °C.

Calibration curves were constructed using alanine, citric acid, fumaric acid, glutamic acid, glycine, lactic acid, malic acid, 2-hydroxybutyric acid, 3-hydroxybutyric acid, linoleic acid, oleic acid, palmitic acid, stearic acid, cholesterol, fructose, glutamine, indole-3-propionic acid, isoleucine, leucine, proline, succinic acid, valine, asparagine, aspartic acid, arachidonic acid, glycerol-3-phosphate, lysine, methionine, ornithine, phenylalanine, serine and threonine purchased from Sigma-Aldrich (St. Louis, MO, USA) at concentration range of 0.1 to 80 µg/mL. An aliquot of each sample was collected and pooled and used as quality control samples, together with a NIST SRM 1950 serum sample and an in-house pooled serum sample.

Relative standard deviations (% RSDs) of the metabolite concentrations in control serum samples showed % RSDs within accepted analytical limits at averages of 27.2% and 29.2% for in-house QC and pooled QC samples.

Statistical methods

All analysis was carried out in the R statistical programming language environment [4]. Missing values in the lipidomic / polar metabolite data were replaced with imputed half-minimums for their respective feature, log2 transformed and scaled to zero mean and unit variance. The sample intersection between the lipidomic, polar metabolomic and clinical information datasets was retained.

Lipidomics and polar metabolite data was clustered using gaussian finite mixture modelling, with the number of clusters (*k*) selected by the model with the highest Bayesian Information Criterion (BIC) (*mclust* package for R, version 5.4.1 [5]).

Cluster variables (CVs) were generated as the mean value of each cluster's member lipids for that sample *s*, giving an *s x k* matrix of values.

Spearman correlation was calculated pairwise between all lipid clusters (LCs), polar metabolites and clinical variables (*corrplot* R package version 0.84) [6]).

Partial correlation network generation

Non-rejection rates (NRRs) of the aforementioned correlations were calculated (*qpNrr* R function, *qpgraph* R package (version 2.16.0) [7], default parameters.) The distribution of NRRs was used to ascertain a conservative cutoff to remove spurious associations (NRR <0.4). The *Rgraphviz* R package (version 2.26.0) [8] was used with customized nodes and edges to project the remaining, non-spurious associations, their directions strengths between all variables as a putative NAFLD interaction network.

Lipids and polar metabolites associated with NAFLD fibrosis

ANOVA and Tukey's honest significant difference (TukeyHSD) tests were performed on the levels all individual lipids and polar metabolites across (1) steatosis grades (S0-S3), (2) between NAFL and NASH diagnoses and (3) across fibrosis stages (F0-F4), with a significance threshold set at $p < 0.05$. Median levels of all lipids and polar metabolites were also calculated for each of these groups for steatosis, NAFL/NASH and fibrosis. For fibrosis and steatosis, correlation was calculated between these median values for each lipid/metabolite and the pathology score itself, to estimate the association between each feature (as a median for the group) and the pathology score itself.

Shared and sex-specific lipids and metabolites across disease perspectives

Data were partitioned into male and female for separate full runs of the analysis pipeline. The results were compared as regards which lipids/metabolites changes significantly and their direction, exactly as for the prior "both-sexes" analysis. Those lipids/metabolites which significantly differ in level across severity of fibrosis or steatosis or between NAFL/NASH were recorded exactly as before (ANOVA+TukeyHSD). The overlaps and differences between male, female and the "both-sexes" analyses were calculated, with overlap being highly strictly limited to those lipids and metabolites which the sexes agreed upon both in terms of having significantly different levels at different severity points, but also in the direction of that change; anything else was deemed disagreement.

Machine learning to assess ability of lipids/metabolites to discern metabolic transition-points in NAFLD evolution

Two binary classification tasks were undertaken by random forest (RF) to stratify patients between (1) $\text{LOW}_{(\text{F0-F1})}$ *vs.* $\text{HIGH}_{(\text{F2-F4})}$ fibrosis scores, and (2) $\text{LOW}_{(\text{F0-F2})}$ *vs.* $\text{HIGH}_{(\text{F3-F4})}$ fibrosis scores (*randomForest* R package (version 4.6-14) for R [9] (70% training, 30% validation data split, downsampled without replacement to the size of the smallest class. Three predictor datasets were

defined; lipidomics data (L), polar metabolites data (M) and a set of clinical variables (C) currently used for NAFLD stratification (including those variables used for calculating gold-standard the FIB-4 score [10]). Minimal feature sets were found through recursive feature addition, guided by model performance (as median AUC) for each number of features used, in decreasing order of feature importance as calculated by a prior run of the random forest using all possible features as a guide. Model performance was assessed for both classification tasks using the three predictor datasets for each, for a total of 6 models constructed and assessed.

Supplementary figures

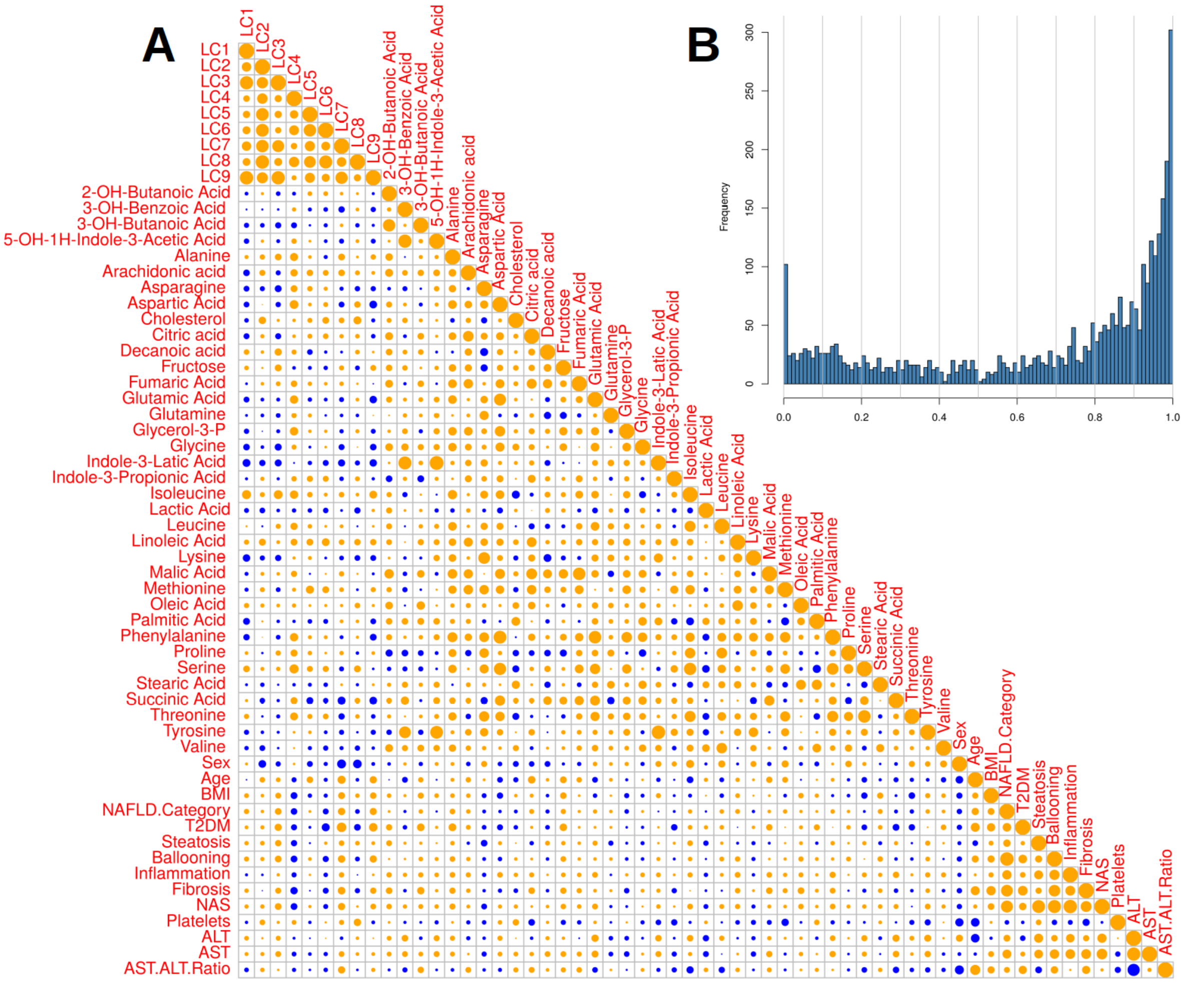

Fig. S1. (A) Spearman Correlation plot showing associations between lipid clusters (LCs), metabolites and clinical data. Orange circles denote positive correlation, blue circles denote negative correlation. Size of circle represents magnitude of correlation. (**B**) Inset histogram showing distribution of all non-rejection rates (NRRs) calculated from correlation plot in panel **A**. NRRs range between 0 (non-spurious) to 1 (likely spurious) associations.

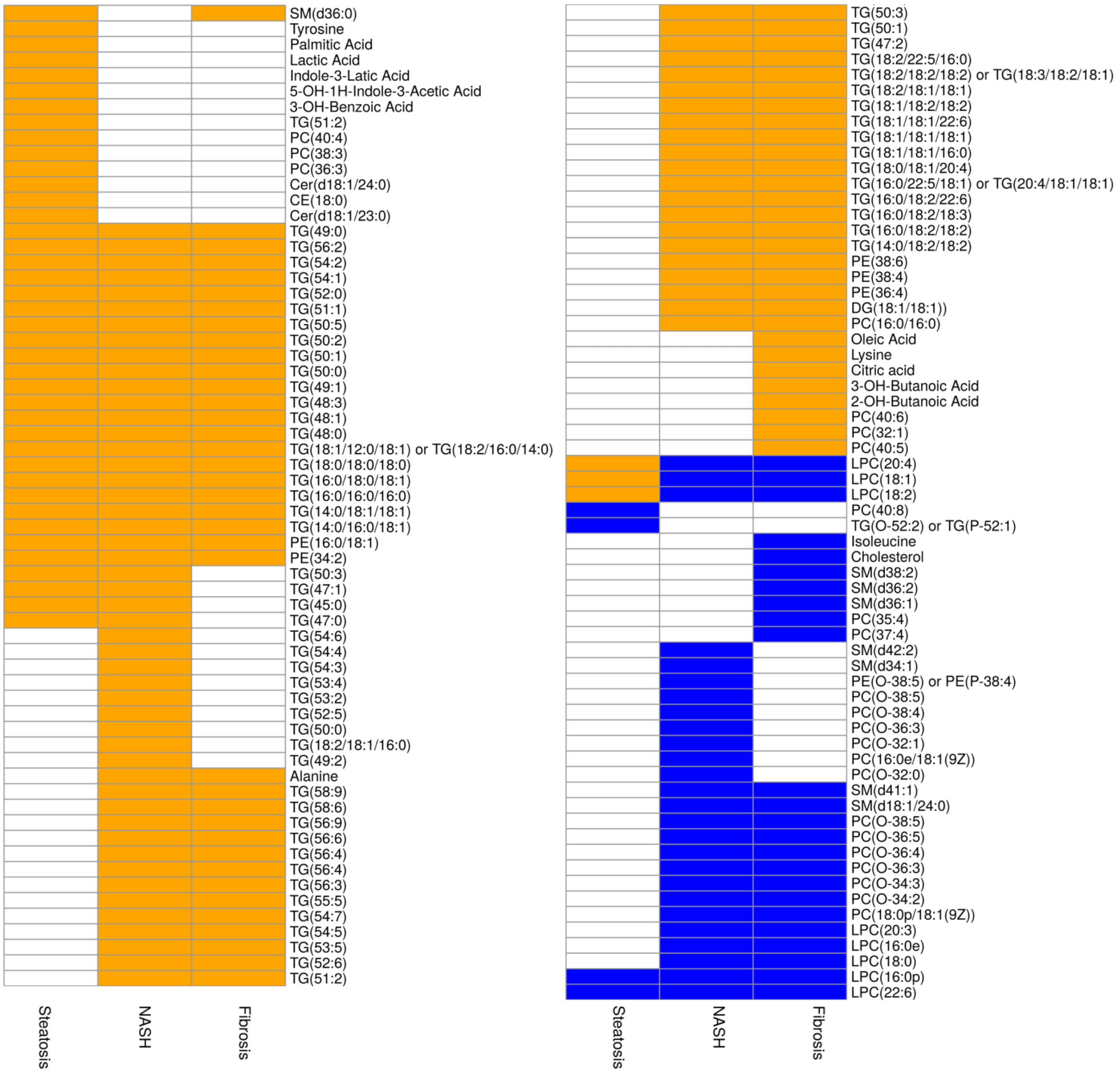

Fig. S2. Feature-clustered heatmap of the trend of direction of change of those lipids and polar metabolites achieving significance ($p < 0.05$) in any of the ANOVA/TukeyHSD analyses across either steatosis, NAFL vs NASH or fibrosis groups. The trend of direction of change is calculated as the sum, across progression S0 to S3, NAFL to NASH and F0 to F4 for steatosis, NASH and fibrosis respectively, of all samples in that group for that lipid / polar metabolite. Lipids / polar metabolites tending to significantly increase across the progression of steatosis / NASH / fibrosis are shown in orange, those that tend to decrease are shown in blue; white for no significant change.

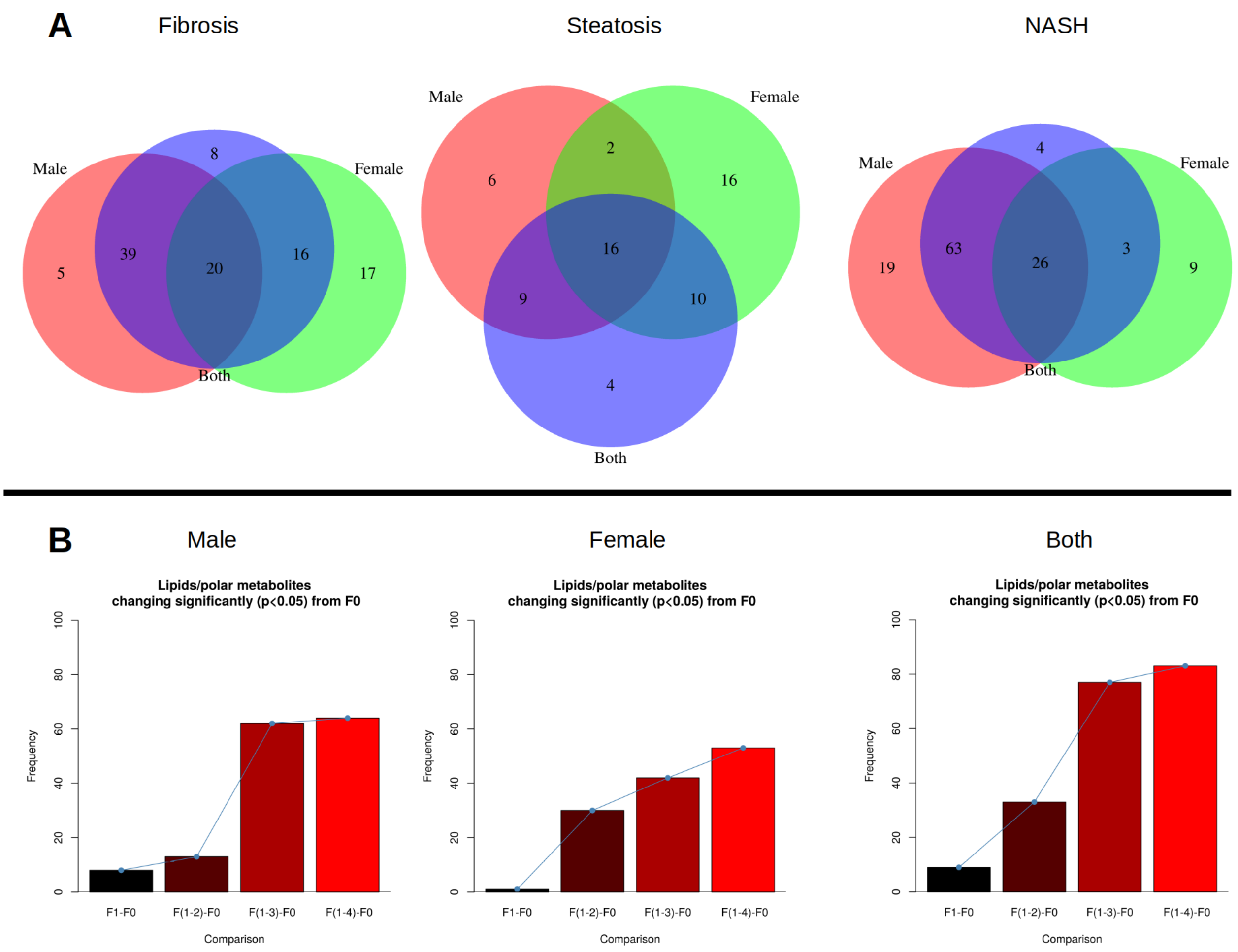

Fig. S3. (A) Venn diagrams depicting the overlap of those lipids/metabolites found to have a significantly-different level across severity scores for fibrosis (F0-F4), steatosis (S0-S3) and NAFL-NASH, when the dataset includes only male subjects (red), only female subjects (green), or both male and female subjects (blue overlay). In order to qualify as "agreement" between any two datasets, a given lipid/metabolite must not only achieve significant change across severity scores, but the direction of that change (estmiated as the sum of changes from the lowest severity score) must agree also. **(B)** Barplots depicting the number of lipids/metabolites found to have a significant change in their level across severity scores of fibrosis. Starting at F0 and progressing through to F4, a significantly-different level of a given lipid/metabolite is only counted towards the lowest severity score at which it changes, so as to avoid counting that lipid/metabolite more than once and inflating the counts in later severity stages.

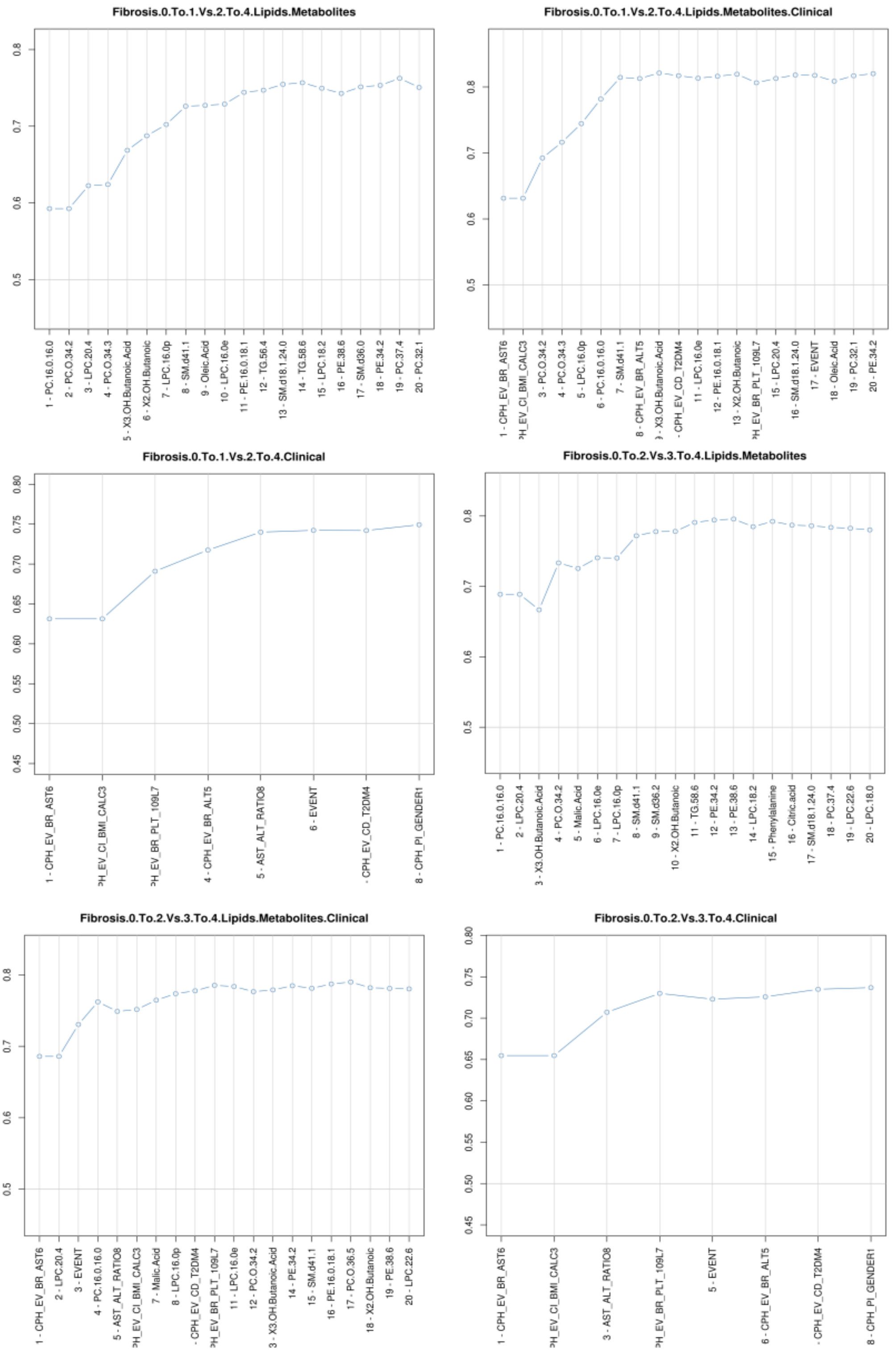

Fig. S4. Recursive feature addition for all 6 machine learning analyses (n = 2 classification tasks, n = 3 data input options, see methods), showing median AUC of models (y axis) as models are built with increasing numbers of features in descending order of importance as determined by prior random forest run.

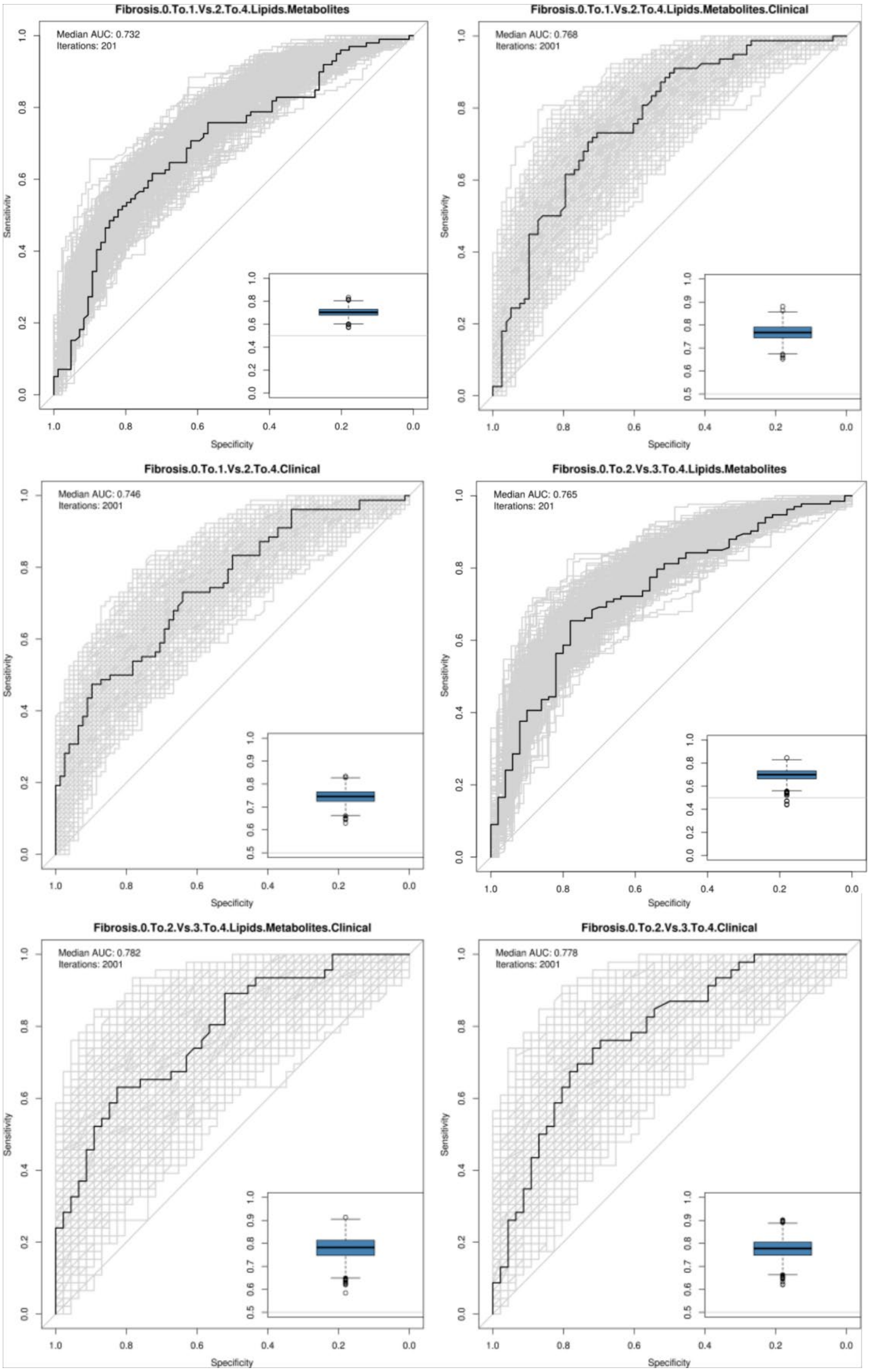

Fig. S5. ROC curves (grey lines) for all 6 analyses (n = 2 classification tasks, n = 3 data input options), with the ROC curve giving median AUC plotted superimposed in black. Inset panel in lower right provides a boxplot to show distribution of AUCs for that task and data input option.

Supplementary tables

All Supplementary Tables are uploaded separately as Excel files. Below are table descriptions.

Table S1. Supplementary table 1 contains three sheets. The “Spearman.Correlations” sheet provides the Spearman correlations between the preprocessed values for all study variables, including lipid clusters (LCs), polar metabolites and clinical variables. The “NRRs” sheet contains the non-rejection rates (NRRs) (cutoff < 0.4) calculated for all of the aforementioned associations as quantified by Spearman correlation in the “Spearman.Correlations” sheet, as calculated by the qpNrr function (see methods). The “Filtered.Correlations” sheet is the result of applying a mask to the Spearman.Correlations sheet, thereby removing likely spurious associations and leaving behind only those satisfying NRR <0.4.

Table S2. Supplementary table 2 lists all lipids and polar metabolites assayed, and provides flags (0,1) (TRUE,FALSE) for whether or not each individual lipid / polar metabolite was found to change significantly (ANOVA/TukeyHSD test) across the scores of fibrosis (0-4), steatosis (0-3) or between NAFL and NASH diagnoses in the clinical data. If a significant change occurred either using ANOVA across all scores, or between at least one score and another (TukeyHSD) for that feature, then this is counted as true. Key: F – fibrosis, S – steatosis, N – NAFL/NASH. The sheets in this spreadsheet file names “Fibrosis”, “Steatosis” and “NASH” provide the values calculated for both ANOVA and inter-group TukeyHSD analyses for all lipid and polar metabolites. Column names which are in the format of two numbered scores (e.g., “2-3”) denote the TukeyHSD comparison performed. These sheets also detail the summarised direction of change of lipids/metabolites, taken as the direction of the summed medians of their individual values.

Table S3. Supplementary table 3 contains multiple sheets, one for both sexes, and one each for male and female summarized analyses. Herein is provided lists of all lipids and metabolites and whether they change (as flags – 0 for FALSE, 1 for TRUE) significantly across fibrosis scores,

steatosis scores or between NAFL/NASH, along with the overall change in direction (increase/decrease as +1/-1, respectively) as a summed score of median changes across severity scores. For overlapping signature characterization, where lipids/metabolites change significantly in the same overall direction in more than one disease perspective (fibrosis, steatosis, NAFL/NASH), flags are given here for the overlaps (again as 0 for FALSE, 1 for TRUE). This is done for all three analyses; both sexes, male only and female only.

Table S4. Supplementary table 4 contains 6 sheets, one containing calculated variable importances for each of the machine learning tasks performed. Each one is named after the classification task, e.g., "F0-1 vs F2-F4 LM" denotes the variable importances of the task seeking to classify between fibrosis scores 0 to 1, versus fibrosis scores 2 to 4, using lipids (L) and polar metabolites (M). The clinical dataset is referred to with (C). The values are median-AUC-scaled values across all 2001 iterations of the initial random forest runs per task, with access to all features, for use in subsequent building of the minimal feature sets.

Supplementary references

[1] McGlinchey A, Sinioja T, Lamichhane S, Sen P, Bodin J, Siljander H, et al. Prenatal exposure to perfluoroalkyl substances modulates neonatal serum phospholipids, increasing risk of type 1 diabetes. Environ Int 2020;143:105935.
[2] Pluskal T, Castillo S, Villar-Briones A, Oresic M. MZmine 2: modular framework for processing, visualizing, and analyzing mass spectrometry-based molecular profile data. BMC Bioinformatics 2010;11:395.
[3] Castillo S, Mattila I, Miettinen J, Oresic M, Hyotylainen T. Data Analysis Tool for Comprehensive Two-Dimensional Gas Chromatography/Time-of-Flight Mass Spectrometry. Analytical Chemistry 2011;83:3058-3067.
[4] R Core Team RFfSC, Vienna, Austria. R: A Language and Environment for Statistical Computing. 2017.
[5] Scrucca L, Fop M, Murphy TB, Raftery AE. mclust 5: Clustering, Classification and Density Estimation Using Gaussian Finite Mixture Models. R j 2016;8:289-317.
[6] Simko TWaV. R package "corrplot": Visualization of a Correlation Matrix. 2017.
[7] Tur I, Roverato A, Castelo R. Mapping eQTL Networks with Mixed Graphical Markov Models. Genetics 2014;198:1377.
[8] Sarkar KDHaJGaLLaRGaSFaFHaD. Rgraphviz: Provides plotting capabilities for R graph objects (Version 2.26.0). 2018.
[9] Liaw A, Wiener M. Classification and Regression by RandomForest. Forest 2001;23.
[10] Sterling RK, Lissen E, Clumeck N, Sola R, Correa MC, Montaner J, et al. Development of a simple noninvasive index to predict significant fibrosis in patients with HIV/HCV coinfection. Hepatology (Baltimore, Md) 2006;43:1317-1325.
