# Supplementary material for: Metabolic signatures across the full spectrum of non-alcoholic fatty liver disease
Source: JHEP Rep. 2022 Mar 26;4(5):100477. doi: 10.1016/j.jhepr.2022.100477 (PMC9006858; doi:10.1016/j.jhepr.2022.100477)
Supplement: Multimedia component 6 [file mmc6.docx]

**Journal of Hepatology**

**CTAT methods**

- 1. **Biological samples**

| **Description** | **Source** | **Identifier** |
| --- | --- | --- |
| Serum samples from the European NAFLD Registry | Newcastle University | NA |

- 1. **Deposited data**

| **Name of repository** | **Identifier** | **Link** |
| --- | --- | --- |
| Metabolomics Workbench | <http://dx.doi.org/10.21228/M85976> | [*https://www.metabolomicsworkbench.org/*](https://www.metabolomicsworkbench.org/) |

- 1. **Software**

| **Software name** | **Manufacturer** | **Version** |
| --- | --- | --- |
| MZmine | <http://mzmine.github.io/> | 2.52 |
| R | <https://cran.r-project.org/> | 3.6.0 |
|  |  |  |
|  |  |  |
|  |  |  |

- 1. **Other (standard compounds for metabolomics)**

| **Compound** | **Manufacturer** | **CAS number(other identifier** |
| --- | --- | --- |
| 1-(1Z-octadecanyl)-2-(9Z-octadecenoyl)- sn-glycero-3-phosphocholine (PC(18:0p/18:1(9Z))) | Avanti Polar Lipids | [799268-63-6](https://www.sigmaaldrich.com/SE/en/search/799268-63-6?focus=products&page=1&perPage=30&sort=relevance&term=799268-63-6&type=cas_number) |
| 1-(9Z-octadecenoyl)-sn-glycero-3- phosphoethanolamine (LPE(18:1)) | Avanti Polar Lipids | [89576-29-4](https://www.sigmaaldrich.com/SE/en/search/89576-29-4?focus=products&page=1&perPage=30&sort=relevance&term=89576-29-4&type=cas_number) |
| 1-heptadecanoyl-2-hydroxy-sn-glycero-3- phosphocholine (LPC(17:0)) | Avanti Polar Lipids | [59491-62-2](https://www.sigmaaldrich.com/SE/en/search/59491-62-2?focus=products&page=1&perPage=30&sort=relevance&term=59491-62-2&type=cas_number) |
| 1-Hexadecanoyl-2-oleoyl-sn-glycero-3- phosphocholine (PC(16:0/18:1) | Avanti Polar Lipids | [26853-31-6](https://www.sigmaaldrich.com/SE/en/search/26853-31-6?focus=products&page=1&perPage=30&sort=relevance&term=26853-31-6&type=cas_number) |
| 1-Palmitoyl-2-Hydroxy-sn-Glycero-3- Phosphatidylcholine (LPC(16:0)) | Avanti Polar Lipids | [17364-16-8](https://www.sigmaaldrich.com/SE/en/search/17364-16-8?focus=products&page=1&perPage=30&sort=relevance&term=17364-16-8&type=cas_number) |
| 1-palmitoyl-2-oleoyl-sn-glycero-3- phosphoethanolamine (PE(16:0/18:1)) | Avanti Polar Lipids | [26662-94-2](https://www.sigmaaldrich.com/SE/en/search/26662-94-2?focus=products&page=1&perPage=30&sort=relevance&term=26662-94-2&type=cas_number) |
| 1-palmitoyl-d31-2-oleoyl-sn-glycero-3- phosphocholine (PC(16:0/d31/18:1)) | Avanti Polar Lipids | [179093-76-6](https://www.sigmaaldrich.com/SE/en/search/179093-76-6?focus=products&page=1&perPage=30&sort=relevance&term=179093-76-6&type=cas_number) |
| 1-stearoyl-2-hydroxy-sn-glycero-3- phosphocholine (LPC(18:0)) | Avanti Polar Lipids | [19420-57-6](https://www.sigmaaldrich.com/SE/en/search/19420-57-6?focus=products&page=1&perPage=30&sort=relevance&term=19420-57-6&type=cas_number) |
| 1-stearoyl-2-linoleoyl-sn-glycerol (DG(18:0/18:2)) | Avanti Polar Lipids | [34487-26-8](https://www.sigmaaldrich.com/SE/en/search/34487-26-8?focus=products&page=1&perPage=30&sort=relevance&term=34487-26-8&type=cas_number) |
| 2-diheptadecanoyl-sn-glycero-3- phosphocholine (PC(17:0/17:0)) | Avanti Polar Lipids | [70897-27-7](https://www.sigmaaldrich.com/SE/en/search/70897-27-7?focus=products&page=1&perPage=30&sort=relevance&term=70897-27-7&type=cas_number) |
| 2-diheptadecanoyl-sn-glycero-3- phosphoethanolamine (PE(17:0/17:0)) | Avanti Polar Lipids | 26662-94-2 |
| 2-Dioctadecanoyl- -sn-glycero-3- phosphocholine (PC(18:0/18:0)) | Avanti Polar Lipids | [816-94-4](https://www.sigmaaldrich.com/SE/en/search/816-94-4?focus=products&page=1&perPage=30&sort=relevance&term=816-94-4&type=cas_number) |
| N-(9Z-octadecenoyl)-sphinganine (Cer(d18:0/18:1(9Z))) | Avanti Polar Lipids | [34227-83-3](https://www.sigmaaldrich.com/SE/en/search/34227-83-3?focus=products&page=1&perPage=30&sort=relevance&term=34227-83-3&type=cas_number) |
| N-heptadecanoyl-D-erythro- sphingosylphosphorylcholine (SM(d18:1/17:0)) | Avanti Polar Lipids | [121999-64-2](https://www.sigmaaldrich.com/SE/en/search/121999-64-2?focus=products&page=1&perPage=30&sort=relevance&term=121999-64-2&type=cas_number) |
| N-heptadecanoyl-D-erythro-sphingosine (Cer(d18:1/17:0)) | Avanti Polar Lipids | [67492-16-4](https://www.sigmaaldrich.com/SE/en/search/67492-16-4?focus=products&page=1&perPage=30&sort=relevance&term=67492-16-4&type=cas_number) |
| Glycolitocholic acid | Calbiochem (Gibbstown, NJ, U.S.A) | 863-57-0 |
| Glycoursodeoxycholic acid | Calbiochem (Gibbstown, NJ, U.S.A) | 16409-34-0 |
| Glycodeoxycholic acid | Fluka (Buchs, Switzerland) | 16409-34-0 |
| Ursodeoxycholic acid | Fluka (Buchs, Switzerland) | 128-13-2 |
| 3-trioctadecanoylglycerol (TG(18:0/18:0/18:0)) | Larodan | [555-43-1](https://www.larodan.com/product/tristearin/) |
| 3β-Hydroxy-5-cholestene-3-linoleate (ChoE(18:2)) | Larodan | 604-33-1 |
| 3β-hydroxy-5-cholestene-3-stearate (ChoE(18:0)) | Larodan | [35602-69-8](https://www.larodan.com/product/cholesteryl-stearate/) |
| triheptadecanoylglycerol (TG(17:0/17:0/17:0)) | Larodan | [620-67-7](https://www.larodan.com/product/triheptanoin/) |
| trihexadecanoalglycerol (TG(16:0/16:0/16:0)) | Larodan | 555-44-2 |
| 3-hydroxybutanoic acid | Sigma-Aldrich (St. Luis, MO, USA) | [206-099-9](https://www.sigmaaldrich.com/SE/en/search/206-099-9?focus=products&page=1&perPage=30&sort=relevance&term=206-099-9&type=egec_number) |
| alanine | Sigma-Aldrich (St. Luis, MO, USA) | [56-41-7](https://www.sigmaaldrich.com/SE/en/search/56-41-7?focus=products&page=1&perPage=30&sort=relevance&term=56-41-7&type=cas_number) |
| arachidonic acid | Sigma-Aldrich (St. Luis, MO, USA) | [506-32-1](https://www.sigmaaldrich.com/SE/en/search/506-32-1?focus=products&page=1&perPage=30&sort=relevance&term=506-32-1&type=cas_number) |
| arginine | Sigma-Aldrich (St. Luis, MO, USA) | [74-79-3](https://www.sigmaaldrich.com/SE/en/search/74-79-3?focus=products&page=1&perPage=30&sort=relevance&term=74-79-3&type=cas_number) |
| ascorbic acid | Sigma-Aldrich (St. Luis, MO, USA) | [50-81-7](https://www.sigmaaldrich.com/SE/en/search/50-81-7?focus=products&page=1&perPage=30&sort=relevance&term=50-81-7&type=cas_number) |
| asparagine | Sigma-Aldrich (St. Luis, MO, USA) | [5794-13-8](https://www.sigmaaldrich.com/SE/en/search/5794-13-8?focus=products&page=1&perPage=30&sort=relevance&term=5794-13-8&type=cas_number) |
| aspartic acid | Sigma-Aldrich (St. Luis, MO, USA) | [56-84-8](https://www.sigmaaldrich.com/SE/en/search/56-84-8?focus=products&page=1&perPage=30&sort=relevance&term=56-84-8&type=cas_number) |
| cholesterol | Sigma-Aldrich (St. Luis, MO, USA) | [57-88-5](https://www.sigmaaldrich.com/SE/en/search/57-88-5?focus=products&page=1&perPage=30&sort=relevance&term=57-88-5&type=cas_number) |
| citric acid | Sigma-Aldrich (St. Luis, MO, USA) | [77-92-9](https://www.sigmaaldrich.com/SE/en/search/77-92-9?focus=products&page=1&perPage=30&sort=relevance&term=77-92-9&type=cas_number) |
| cysteine | Sigma-Aldrich (St. Luis, MO, USA) | [7048-04-06](https://www.sigmaaldrich.com/SE/en/search/7048-04-6?focus=products&page=1&perPage=30&sort=relevance&term=7048-04-6&type=cas_number) |
| decanoic acid | Sigma-Aldrich (St. Luis, MO, USA) | [334-48-5](https://www.sigmaaldrich.com/SE/en/search/334-48-5?focus=products&page=1&perPage=30&sort=relevance&term=334-48-5&type=cas_number) |
| fructose | Sigma-Aldrich (St. Luis, MO, USA) | [57-48-7](https://www.sigmaaldrich.com/SE/en/search/57-48-7?focus=products&page=1&perPage=30&sort=relevance&term=57-48-7&type=cas_number) |
| fructose-6-phosphate | Sigma-Aldrich (St. Luis, MO, USA) | [103213-47-4](https://www.sigmaaldrich.com/SE/en/search/103213-47-4?focus=products&page=1&perPage=30&sort=relevance&term=103213-47-4&type=cas_number) |
| fumaric acid | Sigma-Aldrich (St. Luis, MO, USA) | [110-17-8](https://www.sigmaaldrich.com/SE/en/search/110-17-8?focus=products&page=1&perPage=30&sort=relevance&term=110-17-8&type=cas_number) |
| glucose-6-phosphate | Sigma-Aldrich (St. Luis, MO, USA) | [3671-99-6](https://www.sigmaaldrich.com/SE/en/search/3671-99-6?focus=products&page=1&perPage=30&sort=relevance&term=3671-99-6&type=cas_number) |
| glutamic acid | Sigma-Aldrich (St. Luis, MO, USA) | [56-86-0](https://www.sigmaaldrich.com/SE/en/search/56-86-0?focus=products&page=1&perPage=30&sort=relevance&term=56-86-0&type=cas_number) |
| glutamine | Sigma-Aldrich (St. Luis, MO, USA) | [56-85-9](https://www.sigmaaldrich.com/SE/en/search/56-85-9?focus=products&page=1&perPage=30&sort=relevance&term=56-85-9&type=cas_number) |
| glyceraldehyde | Sigma-Aldrich (St. Luis, MO, USA) | [453-17-8](https://www.sigmaaldrich.com/SE/en/search/453-17-8?focus=products&page=1&perPage=30&sort=relevance&term=453-17-8&type=cas_number) |
| glyceraldehyde-3-phosphate | Sigma-Aldrich (St. Luis, MO, USA) | [591-59-3](https://www.sigmaaldrich.com/SE/en/search/591-59-3?focus=products&page=1&perPage=30&sort=relevance&term=591-59-3&type=cas_number) |
| glycerol-3-phosphate | Sigma-Aldrich (St. Luis, MO, USA) | 17989-41-2 |
| glycine | Sigma-Aldrich (St. Luis, MO, USA) | [56-40-6](https://www.sigmaaldrich.com/SE/en/search/56-40-6?focus=products&page=1&perPage=30&sort=relevance&term=56-40-6&type=cas_number) |
| homocysteine | Sigma-Aldrich (St. Luis, MO, USA) | [6027-13-0](https://www.sigmaaldrich.com/SE/en/search/6027-13-0?focus=products&page=1&perPage=30&sort=relevance&term=6027-13-0&type=cas_number) |
| indole-3-acetic acid | Sigma-Aldrich (St. Luis, MO, USA) | [87-51-4](https://www.sigmaaldrich.com/SE/en/search/87-51-4?focus=products&page=1&perPage=30&sort=relevance&term=87-51-4&type=cas_number) |
| indole-3-lactic acid | Sigma-Aldrich (St. Luis, MO, USA) | [832-97-3](https://www.sigmaaldrich.com/SE/en/search/832-97-3?focus=products&page=1&perPage=30&sort=relevance&term=832-97-3&type=cas_number) |
| indole-3-propionic acid | Sigma-Aldrich (St. Luis, MO, USA) | [830-96-6](https://www.sigmaaldrich.com/SE/en/search/830-96-6?focus=products&page=1&perPage=30&sort=relevance&term=830-96-6&type=cas_number) |
| lactic acid | Sigma-Aldrich (St. Luis, MO, USA) | [50-21-5](https://www.sigmaaldrich.com/SE/en/search/50-21-5?focus=products&page=1&perPage=30&sort=relevance&term=50-21-5&type=cas_number) |
| leucine | Sigma-Aldrich (St. Luis, MO, USA) | [61-90-5](https://www.sigmaaldrich.com/SE/en/search/61-90-5?focus=products&page=1&perPage=30&sort=relevance&term=61-90-5&type=cas_number) |
| linoleic acid | Sigma-Aldrich (St. Luis, MO, USA) | [60-33-3](https://www.sigmaaldrich.com/SE/en/search/60-33-3?focus=products&page=1&perPage=30&sort=relevance&term=60-33-3&type=cas_number) |
| lysine | Sigma-Aldrich (St. Luis, MO, USA) | [657-27-2](https://www.sigmaaldrich.com/SE/en/search/657-27-2?focus=products&page=1&perPage=30&sort=relevance&term=657-27-2&type=cas_number) |
| malic acid | Sigma-Aldrich (St. Luis, MO, USA) | [6915-15-7](https://www.sigmaaldrich.com/SE/en/search/6915-15-7?focus=products&page=1&perPage=30&sort=relevance&term=6915-15-7&type=cas_number) |
| methionine | Sigma-Aldrich (St. Luis, MO, USA) | [63-68-3](https://www.sigmaaldrich.com/SE/en/search/63-68-3?focus=products&page=1&perPage=30&sort=relevance&term=63-68-3&type=cas_number) |
| octanoic acid | Sigma-Aldrich (St. Luis, MO, USA) | [124-07-2](https://www.sigmaaldrich.com/SE/en/search/124-07-2?focus=products&page=1&perPage=30&sort=relevance&term=124-07-2&type=cas_number) |
| oleic acid | Sigma-Aldrich (St. Luis, MO, USA) | [112-80-1](https://www.sigmaaldrich.com/SE/en/search/112-80-1?focus=products&page=1&perPage=30&sort=relevance&term=112-80-1&type=cas_number) |
| ornithine | Sigma-Aldrich (St. Luis, MO, USA) | [3184-13-2](https://www.sigmaaldrich.com/SE/en/search/3184-13-2?focus=products&page=1&perPage=30&sort=relevance&term=3184-13-2&type=cas_number) |
| palmitic acid | Sigma-Aldrich (St. Luis, MO, USA) | [1957-10-03](https://www.sigmaaldrich.com/SE/en/search/57-10-3?focus=products&page=1&perPage=30&sort=relevance&term=57-10-3&type=cas_number) |
| phenylalanine | Sigma-Aldrich (St. Luis, MO, USA) | [63-91-2](https://www.sigmaaldrich.com/SE/en/search/63-91-2?focus=products&page=1&perPage=30&sort=relevance&term=63-91-2&type=cas_number) |
| phosphoenolpyruvate | Sigma-Aldrich (St. Luis, MO, USA) | [4265-07-0](https://www.sigmaaldrich.com/SE/en/search/4265-07-0?focus=products&page=1&perPage=30&sort=relevance&term=4265-07-0&type=cas_number) |
| proline | Sigma-Aldrich (St. Luis, MO, USA) | [147-85-3](https://www.sigmaaldrich.com/SE/en/search/147-85-3?focus=products&page=1&perPage=30&sort=relevance&term=147-85-3&type=cas_number) |
| ribose-5-phosphate | Sigma-Aldrich (St. Luis, MO, USA) | [207671-46-3](https://www.sigmaaldrich.com/SE/en/search/207671-46-3?focus=products&page=1&perPage=30&sort=relevance&term=207671-46-3&type=cas_number) |
| serine | Sigma-Aldrich (St. Luis, MO, USA) | [56-45-1](https://www.sigmaaldrich.com/SE/en/search/56-45-1?focus=products&page=1&perPage=30&sort=relevance&term=56-45-1&type=cas_number) |
| stearic acid | Sigma-Aldrich (St. Luis, MO, USA) | [1957-11-04](https://www.sigmaaldrich.com/SE/en/search/57-11-4?focus=products&page=1&perPage=30&sort=relevance&term=57-11-4&type=cas_number) |
| succinic acid | Sigma-Aldrich (St. Luis, MO, USA) | [110-15-6](https://www.sigmaaldrich.com/SE/en/search/110-15-6?focus=products&page=1&perPage=30&sort=relevance&term=110-15-6&type=cas_number) |
| threonine | Sigma-Aldrich (St. Luis, MO, USA) | [80-68-2](https://www.sigmaaldrich.com/SE/en/search/80-68-2?focus=products&page=1&perPage=30&sort=relevance&term=80-68-2&type=cas_number) |
| tryptophan | Sigma-Aldrich (St. Luis, MO, USA) | [73-22-3](https://www.sigmaaldrich.com/SE/en/search/73-22-3?focus=products&page=1&perPage=30&sort=relevance&term=73-22-3&type=cas_number) |
| tyrosine | Sigma-Aldrich (St. Luis, MO, USA) | [60-18-4](https://www.sigmaaldrich.com/SE/en/search/60-18-4?focus=products&page=1&perPage=30&sort=relevance&term=60-18-4&type=cas_number) |
| [2D4]-succinic acid | Sigma-Aldrich (St. Luis, MO, USA) | [14493-42-6](https://www.sigmaaldrich.com/SE/en/search/14493-42-6?focus=products&page=1&perPage=30&sort=relevance&term=14493-42-6&type=cas_number) |
| [2D5]-glutamic acid | Sigma-Aldrich (St. Luis, MO, USA) | [108395-13-7](https://www.sigmaaldrich.com/SE/en/search/108395-13-7?focus=products&page=1&perPage=30&sort=relevance&term=108395-13-7&type=cas_number) |
| [2D8]-valine | Sigma-Aldrich (St. Luis, MO, USA) | [203784-63-8](https://www.sigmaaldrich.com/SE/en/search/203784-63-8?focus=products&page=1&perPage=30&sort=relevance&term=203784-63-8&type=cas_number) |
| 1-oleoyl-2-hydroxy-sn-glycero-3- phosphocholine (LPC(18:1)) | Sigma-Aldrich (St. Luis, MO, USA) | [19420-56-5](https://www.sigmaaldrich.com/SE/en/search/19420-56-5?focus=products&page=1&perPage=30&sort=relevance&term=19420-56-5&type=cas_number) |
| 2-hydroxybutanoic acid | Sigma-Aldrich (St. Luis, MO, USA) | [600-15-7](https://www.sigmaaldrich.com/SE/en/search/600-15-7?focus=products&page=1&perPage=30&sort=relevance&term=600-15-7&type=cas_number) |
| valine | Sigma-Aldrich (St. Luis, MO, USA) | [72-18-4](https://www.sigmaaldrich.com/SE/en/search/72-18-4?focus=products&page=1&perPage=30&sort=relevance&term=72-18-4&type=cas_number) |
| 1-(1Z-octadecanyl)-2-(9Z-octadecenoyl)- sn-glycero-3-phosphocholine (PC(18:0p/18:1(9Z))) | Avanti Polar Lipids | [799268-63-6](https://www.sigmaaldrich.com/SE/en/search/799268-63-6?focus=products&page=1&perPage=30&sort=relevance&term=799268-63-6&type=cas_number) |
| 1-(9Z-octadecenoyl)-sn-glycero-3- phosphoethanolamine (LPE(18:1)) | Avanti Polar Lipids | [89576-29-4](https://www.sigmaaldrich.com/SE/en/search/89576-29-4?focus=products&page=1&perPage=30&sort=relevance&term=89576-29-4&type=cas_number) |
| 1-heptadecanoyl-2-hydroxy-sn-glycero-3- phosphocholine (LPC(17:0)) | Avanti Polar Lipids | [59491-62-2](https://www.sigmaaldrich.com/SE/en/search/59491-62-2?focus=products&page=1&perPage=30&sort=relevance&term=59491-62-2&type=cas_number) |
| 1-Hexadecanoyl-2-oleoyl-sn-glycero-3- phosphocholine (PC(16:0/18:1) | Avanti Polar Lipids | [26853-31-6](https://www.sigmaaldrich.com/SE/en/search/26853-31-6?focus=products&page=1&perPage=30&sort=relevance&term=26853-31-6&type=cas_number) |
| 1-Palmitoyl-2-Hydroxy-sn-Glycero-3- Phosphatidylcholine (LPC(16:0)) | Avanti Polar Lipids | [17364-16-8](https://www.sigmaaldrich.com/SE/en/search/17364-16-8?focus=products&page=1&perPage=30&sort=relevance&term=17364-16-8&type=cas_number) |
| 1-palmitoyl-2-oleoyl-sn-glycero-3- phosphoethanolamine (PE(16:0/18:1)) | Avanti Polar Lipids | [26662-94-2](https://www.sigmaaldrich.com/SE/en/search/26662-94-2?focus=products&page=1&perPage=30&sort=relevance&term=26662-94-2&type=cas_number) |
| 1-palmitoyl-d31-2-oleoyl-sn-glycero-3- phosphocholine (PC(16:0/d31/18:1)) | Avanti Polar Lipids | [179093-76-6](https://www.sigmaaldrich.com/SE/en/search/179093-76-6?focus=products&page=1&perPage=30&sort=relevance&term=179093-76-6&type=cas_number) |
| 1-stearoyl-2-hydroxy-sn-glycero-3- phosphocholine (LPC(18:0)) | Avanti Polar Lipids | [19420-57-6](https://www.sigmaaldrich.com/SE/en/search/19420-57-6?focus=products&page=1&perPage=30&sort=relevance&term=19420-57-6&type=cas_number) |
| 1-stearoyl-2-linoleoyl-sn-glycerol (DG(18:0/18:2)) | Avanti Polar Lipids | [34487-26-8](https://www.sigmaaldrich.com/SE/en/search/34487-26-8?focus=products&page=1&perPage=30&sort=relevance&term=34487-26-8&type=cas_number) |
| 2-diheptadecanoyl-sn-glycero-3- phosphocholine (PC(17:0/17:0)) | Avanti Polar Lipids | [70897-27-7](https://www.sigmaaldrich.com/SE/en/search/70897-27-7?focus=products&page=1&perPage=30&sort=relevance&term=70897-27-7&type=cas_number) |

- 1. **Please provide the details of the corresponding methods author for the manuscript:**

| Matej Oresic ([matej.oresic@oru.se](mailto:matej.oresic@oru.se)) |
| --- |
